# The Dutch-translated Climate and Health Tool for nurses: cross-cultural adaptation and validation

**DOI:** 10.1016/j.ijnsa.2026.100603

**Published:** 2026-06-17

**Authors:** Rick R.A. Hendriks, Leontien van Wely, Yara Gutter, Monique Chambon, Nicole Hunfeld, Kim J. Verhaegh

**Affiliations:** a Leiden University of Applied Sciences, Department of Health, Zernikedreef 11, Postbus 382, 2300 AJ, Leiden, the Netherlands; b University of Amsterdam, Department of Psychology, PO Box 15900, 1001 NK Amsterdam, the Netherlands; c Erasmus University Medical Centre, Department of Intensive Care Adults, Dr. Molewaterplein 40, 3015 GD Rotterdam, the Netherlands; d Alrijne Hospital, Department of Alrijne Academy, Simon Smitweg 1, 2353 GA, Leiderdorp, the Netherlands

**Keywords:** Planetary Health, Nursing, Climate change, Psychometric, Public Health, Nursing leadership, Cultural Adaptation

## Abstract

**Background:**

Health systems in the Netherlands must be equipped to respond to climate-related challenges. As a trusted interface between society and healthcare, nurses play a critical leadership role in mitigating and addressing climate-sensitive health risks. Establishing a baseline understanding of nurses' awareness, concern, motivation and behaviors related to climate change is therefore essential. However, no validated Dutch instrument exists to assess these competencies. The Climate and Health Tool, originally developed and validated in the United States of America, has demonstrated strong reliability and validity and offers a promising foundation for adaptation.

**Objective:**

To translate, culturally adapt and validate the Climate and Health Tool from American-English into Dutch.

**Methods:**

The study followed a six-stage process. The first five stages focused on cross-cultural adaptation and translation, including forward-backward translation, expert committee review, and field pre-testing. The sixth stage consisted of psychometric evaluation. Nurses from across the Netherlands were recruited through the Dutch Nurses Association and other in- and external networks, starting from the 19th of June 2025. Data were analyzed using a confirmatory factor analysis. Internal consistency was evaluated using McDonald's omega and Cronbach's alpha.

**Results:**

Translation procedures, expert committee review, and pre-testing interviews indicated good overall comprehensibility, with minor revisions implemented to improve interpretability. Psychometric evaluation was conducted in a sample of 292 nurses with varied ages, work experience and healthcare backgrounds. The 22-item, five-factor Dutch version of the Climate and Health Tool demonstrated moderate model fit, good internal consistency, and acceptable standardized factor loadings.

**Conclusions:**

This study provides a reliable and validated Dutch instrument for assessing nurses' awareness, concern, motivation, and behavior regarding climate change and health. The Dutch version of the Climate and Health Tool offers researchers, educators, and healthcare organizations a robust method for evaluating climate-related competencies and supports broader efforts to prepare Dutch health systems for current and emerging climate-related challenges.

**Tweetable abstract:**

The Dutch version of the Climate and Health Tool is a validated Dutch instrument to assess nurses’ awareness, concern, motivation and behavior on climate related health challenges—supporting stronger climate ready health systems.

**What is already known**

• Nurses play a key leadership role in advancing human and planetary health.
• Studies have identified substantial gaps in climate-related awareness, preparedness, and actions among healthcare professionals, including nurses.
• In the Netherlands, no validated instruments are available to assess nurses' awareness, concern, motivation, and behavior related to climate change and health.

Alt-text: Unlabelled box dummy alt text

**What this paper adds**

• This study introduces a validated Dutch instrument for measuring nurses' awareness, concern, motivations, and behavior regarding climate change and health.
• The Dutch version of the Climate and Health Tool enables nursing research and practice to monitor changes in climate-related competencies over time.
• The Dutch version of the Climate and Health Tool provides a standardized method for identifying gaps in climate-related awareness and action among nurses, supporting targeted interventions, education, and policy development.

Alt-text: Unlabelled box dummy alt text

## 1 Introduction

The global environment is undergoing significant transformations, including rising temperatures, deforestation, and increasing pollution. Since the onset of industrialization, human activity has substantially altered Earth's ecosystems (Planetary Health Alliance, 2026; Vandenberg et al., 2024), a phenomenon described by atmospheric chemist Paul Crutzen as the 'Anthropocene' (Albrecht, 2019). The current era is characterized by humanity's impact on geological and ecological systems (Planetary Health Alliance, 2026). Changes in air quality, water resources, food production, disease dynamics, and overall environmental habitability are already affecting human health, with projections of intensification in the future (Planetary Health Alliance, 2026; USGCRP, 2017).

Analysis of the Netherlands shows that the Dutch healthcare sector contributes 7.3% of the national carbon footprint (Steenmeijer et al., 2022) and that the development or revision of health policies and programs has been limited (World Health Organization, 2020). Concurrently, health impacts of climate change are already observable in the Netherlands. Rising temperatures, prolonged allergy seasons, and deteriorating air quality in urban areas contribute to an increased disease burden (Quarsie et al., 2021; Staatsen et al., 2024). With vulnerable populations being disproportionately affected by these changes, existing health inequities are exacerbated (Eitelwein et al., 2024). These trends may result in sudden increases in hospital admissions, placing additional pressure on healthcare services. To address these challenges, a more integrated and future-oriented perspective is needed: planetary health (Whitmee et al., 2015).

The concept of *planetary health* emphasizes the interdependence between human development and ecological integrity, recognizing that sustainable health outcomes require that we maintain safe environmental limits. This can be done through careful consideration of both human systems (political, economic, and social) and the Earth's natural systems that establish the environmental boundaries within which humanity can thrive (Whitmee et al., 2015).

In this context, health systems must be equipped to address climate-related challenges effectively. This includes enhancing healthcare infrastructure to manage increased patient demand and ensuring that healthcare professionals receive adequate training (Santos et al., 2025). This is especially applicable for professionals operating at the intersection of patient care and public health, as they play a critical leadership role in mitigating and responding to climate-sensitive health risks. Nurses, who represent approximately 57% of the global healthcare occupations, are particularly central to these efforts (World Health Organization, 2025). As the largest professional group within healthcare, they serve as a trusted key interface between health systems and society (International Council of Nurses, 2024; Salvage and White, 2020). Increased and accelerated climate action by nurses through the development and implementation of mitigation and adaptation strategies, policies, and programs is suggested to be pivotal for the future of people's health and the planet (International Council of Nurses, 2024). Additionally, education has a critical role in preparing nursing graduates by giving them the knowledge and skills needed to address the challenge of planetary health effectively (Levett-Jones et al., 2024).

Current evidence indicates gaps in climate-related awareness and action among healthcare professionals, including nurses (Kotcher et al., 2021; Müller et al., 2024; Ryan et al., 2020; Schenk et al., 2021). To enable nurses to promote and integrate the principles of planetary health, it is essential that they first develop awareness and acquire the necessary knowledge and competencies. This can be achieved through evidence-based research, inter- and multidisciplinary collaborations, increasing awareness among nurse educators, integrating climate-change-related content into nursing curricula, strengthening clinical skills, and fostering advocacy and leadership capabilities (Lee et al., 2024; Santos et al., 2025). However, before such actions can be taken, it is crucial to establish a baseline understanding of current nurses' competencies regarding climate change and health, so that nursing education, research, and practice can better align to the needs of nurses. Achieving this requires reliable and valid measurement instruments, but such instruments are not yet available in the Netherlands.

A systematic review by Santos et al. (2024) evaluated the most reliable, robust, and valid instruments for the measurement of nurses' knowledge and awareness of climate change and climate-associated diseases. Among the instruments included in the study, the Climate and Health Tool (abbr. CHANT) presented the best psychometric properties (Santos et al., 2024). This instrument is originally developed and validated in 2018 by Schenk et al. (2019) in the United States of America and has been applied nationally and internationally to assess healthcare professionals' awareness, concern, motivation, and behaviors regarding climate change and health (Winquist et al., 2023). While originally created for nurses, the Climate and Health Tool's strength is that it can also be used to measure other healthcare professionals (Rangel et al., 2024). These strengths are particularly relevant in the Dutch context, as they can support and reinforce ongoing national efforts, such as the Green Deal Sustainable Care initiative (Rijksdienst voor Ondernemend Nederland. 2026) or the BN2030 Dutch nursing curriculum profile (Bouwes et al., 2023), which places increasing emphasis on sustainability and the role of nurses in delivering climate-resilient care.

A cultural adaptation of the Climate and Health Tool is essential to accurately assess Dutch nurses' awareness, concern, motivation, and behaviors related to climate change and health. Cross-cultural adaptation addresses both linguistic and cultural considerations when preparing a questionnaire for use in a new context (Beaton et al., 2000). Its goal is to ensure equivalence between the source and target versions in terms of content relevance and interpretability (Beaton et al., 2000). Successful adaptation requires attention to multiple types of equivalence (semantic, idiomatic, experiential, and conceptual) to ensure that items carry the same meaning and significance across cultures (Beaton et al., 2000; Cruchinho et al., 2024). This study therefore focuses on the systematic cultural adaptation, translation and validation of the Climate and Health Tool from American-English into Dutch.

## 2 Method

### 2.1 Instrument

The Climate and Health Tool is an American valid and reliable, 22-item, 5-factor instrument developed by Schenk et al. (2019) to assess health professionals’ awareness, motivation, and behaviors regarding climate change and health (Schenk et al., 2019; Winquist et al., 2023) (see supplementary file I). The Cronbach's α coefficient is reported to range from 0.67 to 0.91 (Winquist et al., 2023).

The Awareness factor includes five items measuring awareness of the impact of climate change on human health (Cronbach's α = 0.85), with a range of 0 "not at all familiar” to 4 "extremely familiar” (total score 0–20)(Winquist et al., 2023).

The Concern factor includes five items measuring health professionals’ concern about the impacts of climate change on health (Cronbach's α = 0.91) with a range of scores of 0 "not at all" to 4 "extremely" (total score 0–20)(Winquist et al., 2023).

The Motivation factor includes three items measuring health professionals’ motivation to change their personal practice to address climate change (Cronbach's α = 0.91) with a range of scores from 0 "very untrue for me" to 4 "very true for me" (total score 0–12)(Winquist et al., 2023).

Lastly, behavior is divided into two factors: Home behavior and Work behavior. The Home behavior factor includes five items measuring climate-friendly behaviors at home (Cronbach's α = 0.75) with a range of scores from 0 "never" to 4 "always" (total score 0–20)(Winquist et al., 2023). The Work behavior factor includes four items measuring climate-friendly behaviors at work (Cronbach's α = 0.67) with a range from 0 "never" to 4 "always" (total score 0–16)(Winquist et al., 2023).

### 2.2 Procedure

**Fig. 1:**
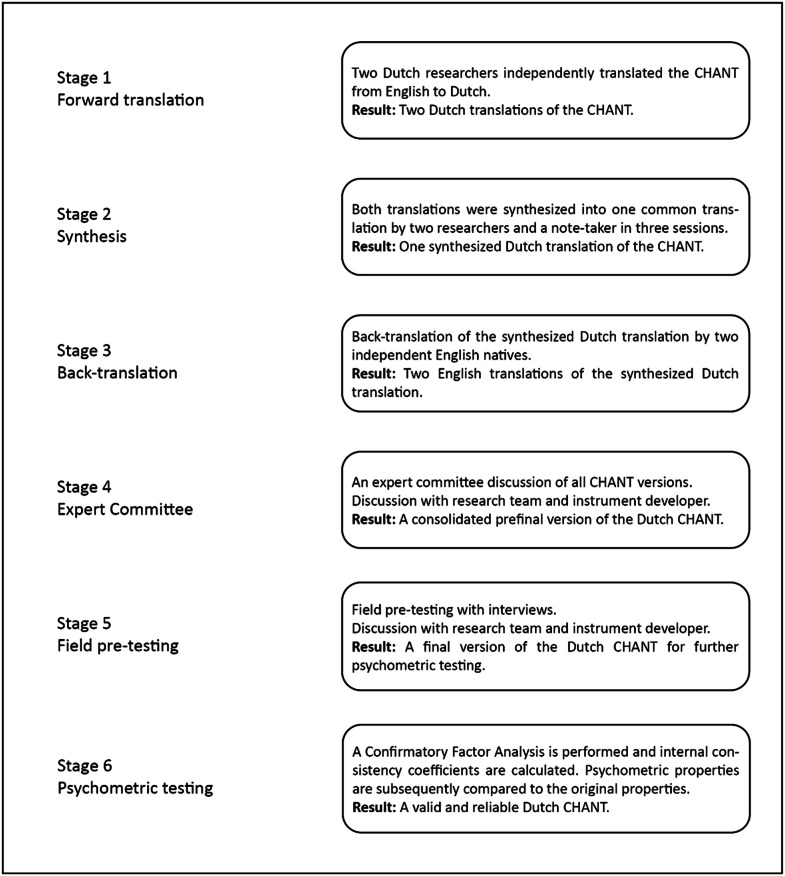
Graphic representation of the cross-adaptation process for the Dutch Climate and Health Tool.

Permission to translate the Climate and Health Tool from English into Dutch was received from the developer by email on the 30th of August 2024. A six-stage cross-cultural adaptation process was then initiated following the guidelines by Beaton et al. (2000). The term "cross-cultural adaptation" is used to encompass a process that looks at both language (translation) and cultural adaptation issues in the process of preparing a questionnaire for use in another setting (Beaton et al., 2000). Important to the success of the adaptation process is the consideration of multiple types of equivalence between the original and the translated instrument (Beaton et al., 2000; Cruchinho et al., 2024). That is, semantic equivalence – the degree to which the meaning of words, phrases or sentences is preserved when adapted to another language; idiomatic equivalence – concerned with the accurate adaptation of idiomatic expressions, colloquialisms, or culturally specific phrases so that they convey the same meaning and tone in the target language; experiential equivalence – the extent to which concepts or experiences described in one culture exist and are understood in another; and conceptual equivalence – the alignment of underlying concepts or constructs across different languages and cultures to ensure that terms and phrases represent the same theoretical or conceptual meaning (Beaton et al., 2000). The guidelines by Beaton et al. (2000) serve as a template for the translation and cultural adaptation process during this study. Fig. 1 outlines the cross-cultural adaptation process that was followed during this study. Any disagreements during the study were resolved through consensus-based discussions with the research team or by discussion with the developer.

#### 2.2.1 Stage 1 – Forward translation

The two Dutch researchers comprised a senior researcher with a background in physical therapy and climate-related health research (L.W.), and a junior researcher with a background in social psychology (R.H.).

#### 2.2.2 Stage 2 - Synthesis

The two Dutch researchers (L.W. & R.H.) discussed differences in both translations and synthesized them into a single version. Note-takers were invited to record the process.

#### 2.2.3 Stage 3 – Back-translation

Back-translations were conducted by a physical therapy lecturer with a British background, and a senior lecturer-researcher with a medical background and certification in ENG-NL translation.

#### 2.2.4 Stage 4 – Expert Committee

The expert committee comprised seven members, five of whom had medical background. The committee included three senior researchers (two of whom held lecturer-researcher positions), two junior researchers (including one registered nurse), one program lead for the university's nursing curriculum, and one professor who is also a registered nurse. The committee discussed all versions of the instrument (supplementary file III) during a two-hour session and consolidated a prefinal version. Subsequently, a list of questions was compiled and discussed with the developer (supplementary file IV).

#### 2.2.5 Stage 5 – Field pre-testing

To assess the comprehensibility, relevance, acceptability, and equivalence to the original instrument, the consolidated prefinal version was tested as an online questionnaire. Nursing students, educators and practitioners of various levels and specializations were invited to participate in the pre-testing stage. Participants were recruited until data saturation was reached. Proficiency in the Dutch language was a requirement for participation. The questionnaire was distributed using Qualtrics (https://www.qualtrics.com), via an anonymous link or QR-code. The questionnaire presented the prefinal version along with the opportunity to provide textual input. This allowed participants to provide feedback regarding the comprehensibility, relevance, and acceptability of the prefinal version. The participants’ insights, suggestions, and feedback were subsequently confirmed and explored in an interview of approximately 30 min. Data collection took place between the 19th of June 2025 and the 8th of September 2025. Interviews were audio-recorded and transcribed. Findings were summarized and discussed within the research team to determine which components of the instrument required adaptation and how these modifications should be implemented. Additionally, a list of questions was compiled and discussed with the developer (supplementary file IV).

#### 2.2.6 Stage 6 – Psychometric evaluation

Psychometric evaluation was conducted to assess the validity and internal consistency properties of the final Dutch version. In collaboration with researchers from the University of Amsterdam and the Erasmus Medical Centre, the final Dutch version was distributed by newsletter amongst members of the Dutch Nurses Association (V&VN). The instrument was administered as an online questionnaire using Castor Electronic Data Capture. To further support recruitment, participants were also approached via existing personal networks and via research networks like the ESCH-R consortium (www.ESCH-R.org) and Greening healthcare together (www.samendezorgvergroenen.nl). A minimum sample size of 220 participants was targeted, based on the recommended ratio of at least 10 participants per item (Kyriazos, 2018). Data collection took place between the 29th of October 2025 and the 5th of January 2026. Demographic and professional information was collected to gain insight into the background of the respondents, however these data were stored separately from the instrument responses to ensure participant anonymity. Psychometric evaluation was conducted using a confirmatory factor analysis of the five-factor model, similar to the original instrument (Winquist et al., 2022). Results were compared to the psychometric properties of the original instrument to ensure equivalence (Winquist et al., 2023). To support the interpretation of the results, and determine whether revisions to the instrument were warranted, a statistical expert was consulted.

### 2.3 Data analysis

#### 2.3.1 Field pre-testing

Interview transcripts were categorized according to the questions in the questionnaire. This included that any information given regarding a particular question was categorized to that particular question. Data was systematically organized and discussed by two researchers (Y.G. & R.H.). Results were discussed by the research team. A list of questions was compiled and discussed with the developer (supplementary file IV).

#### 2.3.2 Psychometric testing

Statistical analyses were conducted in SPSS (IBM, United States of America, version 29.0.0.0) and Rstudio (Posit, United States of America, version 4.5.2). Descriptive statistics were used to summarize participant demographic and professional characteristics. One-sample *t*-tests were applied to test factor mean scores. Statistical assumptions (Kaiser-Meyer-Olkin, Bartlett's test of sphericity, Multicollinearity and Multivariate normality) were checked. A confirmatory factor analysis was conducted to assess construct validity of the five-factor instrument with 22-items, using the R packages Lavaan, Haven, semPlot, and semTools. Similar to the original instrument, the Full Information Maximum Likelihood Robust estimation (FIMLR) was used to estimate data and account for any non-normality and missing data (Byrne, 1994; Brown, 2015; Winquist et al., 2023). Overall model fit was evaluated using several fit indices. Measures of fit included the Root Mean Square Error of Approximation (RMSEA, threshold: ≤ 0.080), Standardized Root Mean Square Residual (SRMR, threshold: ≤ 0.080), Comparative Fit Index (CFI, threshold ≥ 0.90), and Chi-square fit index (threshold: cmin/df = < 2 or < 5) (Hu and Bentler, 1999; Schermelleh-Engel et al., 2003; Hooper et al., 2008; Brown, 2015; McNeish et al., 2018). Internal consistency was evaluated using McDonald's ω, and Cronbach's α similar to the original instrument. McDonald's ω and Cronbach's α above 0.70 indicate positive internal consistency (McDonald, 1999; Tavakol and Dennick, 2011). A factor loading threshold of 0.30 was applied, in line with recommendations by Kline (1994).

### 2.4 Ethical considerations

The study was approved by the Ethics Review Committee of the University of Applied Sciences Leiden (file nr.: 20250516 Hendriks) on the 19th of May 2025 and the Ethics Review Board of the University of Amsterdam on the 23rd of October 2025 (FMG-16088). Research data management is in compliance with the Netherlands Code of Conduct for Research Integrity. Informed consent was given by participants. Data from questionnaires and interviews were pseudonymized to safeguard participants’ privacy and only designated members of the research team had access to identifiable source data. For reporting and analysis, fully anonymized data were used. All data and research documentation are stored securely on the universities' protected servers and will be retained for 10 years. 'Not registered'

## 3 Results

### 3.1 Translation and cross-cultural adaptation

Any institute, organization, statistic, weather event, or disease mentioned in the original instrument was changed to fit the context of the Netherlands. Mentions of ethnicity (e.g. people of color) were largely omitted from the translation, due to cultural differences and the perceived wrongful mention in the same context as homelessness in 'vulnerable populations'. The expert committee uncovered inconsistencies in the initial translated synthesis and back-translations, so textual changes were made to fit the nursing context better and increase similarity with the original tool (supplementary file III). Also, in contrast to the original scores of 0–4, scores from 1–5 were used for this study.

A total of *N* = 11 nurses (*M*^age^ = 37, Gender = 90.9% female) completed the pre-test questionnaire, all of whom participated in the follow-up interviews. The sample comprised *n* = 4 students, *n* = 3 educators, and *n* = 6 practitioners (dual roles were present). Findings revealed minor inconsistencies in word usage, for example in differentiating between 'earth' and 'planet' when talking about changes to the planet. Participants' interpretation of our translation of the word 'community' was varied, which prompted a research team discussion. In addition, illustrative examples were incorporated into items that participants found moderately difficult to interpret. For example, interviews findings revealed that the item: "*I want to prepare for health impacts of climate change at my workplace"* was rather vague, prompting clarification questions such as: *"what is meant by 'preparing my workplace’?"*. Following further explanation from the developer regarding the original intent of the item, a decision was made to include two illustrative examples to enhance clarity and improve interpretability.

### 3.2 Psychometric evaluation

**Fig. 2:**
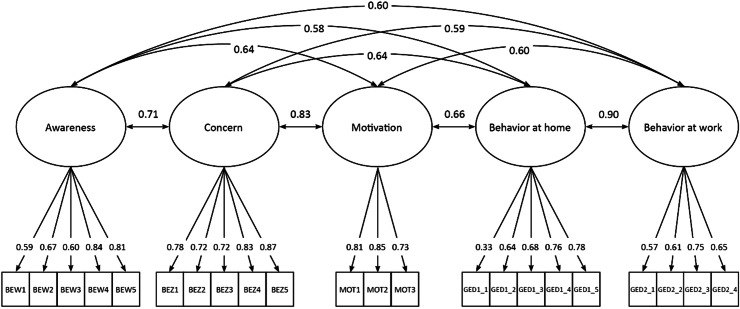
Path diagram for the five-factor Model CHANT. *Note.*Fig. 2 illustrates relationships between the factors (factor correlations) and the underlying items (standardized item loading coefficients) in the Dutch version of the Climate and Health Tool.

Following data cleaning (e.g. removing incomplete questionnaires), the final sample consisted of *N* = 292 nurses. This exceeded the target sample size of *N* = 220. Sample demographic and professional characteristics are presented in Table 1. One-factor *t-*tests conducted on the factor mean scores indicated that all factors differed statistically significant (*p* < .001) from the instrument's midpoint (Awareness: *M* = 3.9, *SD* = 0.77; Concern: *M* = 3.8, *SD* = 0.85; Motivation: *M* = 3.8, *SD* = 0.88; Home behavior: *M* = 3.6, *SD* = 0.67; Work behavior: *M* = 3.3, *SD* = 0.78), indicating higher scores compared to the midpoint (3). An overall Kaiser-Meyer-Olkin value of 0.91 was obtained and Bartlett's test of sphericity gave a statistically significant result (*p* < .001), indicating that the dataset is suitable for factor analysis. Similar to the original instrument, a confirmatory factor analysis was conducted using a five-factor 22-item model. Results indicated a moderate fit for the five-factor model of the Dutch Climate and Health Tool, χ² (199) = 670.906, *p* < .001, CFI = 0.86, RMSEA = 0.090 and SRMR = 0.06, see Table 2. That is, while the thresholds for RMSEA (≤ 0.080) and CFI (≥ 0.90) were not met, the thresholds for χ² (cmin/df = < 2 or < 5) and SRMR (≤ 0.080) were. All five factors demonstrated good reliability, with McDonald's ω ranging between 0.73 to 0.88 and Cronbach's α ranging between 0.73 to 0.89. All standardized item loading coefficients were > 0.30 and are presented alongside item-level descriptives in Table 3. Factor correlations were statistically significant (*p* < .001), ranging from 0.58 to 0.90 (see Fig. 2).

**Table 1:** Demographic and professional characteristics of the respondents.

	*N*	Frequency	%
**Gender**	292		
Male		50	17.1%
Female		242	82.9%
**Age**	292		
0–29 years		55	18.8%
30–39 years		57	19.5%
40–49 years		56	19.2%
50–59 years		76	26.0%
60+ years		48	16.4%
**Work experience**	292		
0–9 years		74	25.3%
10–19 years		67	22.9%
20+ years		151	51.7%
**Current role**	292		
Direct Care/Clinical		246	84.2%
Faculty/Education		11	3.8%
Quality/Research		7	2.4%
Leadership		10	3.4%
Student		4	1.4%
Other		14	4.8%
**Work setting**	292		
Hospital/Acute Care		209	71.6%
Outpatient care		9	3.1%
Long-term care		24	8.2%
Community care		4	1.4%
Mental Health care		16	5.5%
Primary care		3	1.0%
Education/Faculty		6	2.1%
Other		21	7.2%
**Education**	292		
Vocational		82	28.0%
Bachelor		158	54.1%
Master/University		16	5.5%
Other		36	12.3%

**Table 2:** Fit indices of the five-factor CHANT model.

Model	X^2^	*df*	CFI	SRMR	RMSEA (90% CI)
5-factor model	670.91*	199	0.863	0.060	0.090(0.083, 0.098)

Abbreviations: χ^2^ = Chi-square; *df* = Degrees of Freedom; CFI = Comparative Fit Index; SRMR = Standardized Root Mean Square Residuals; RMSEA = Root Mean Square Error Approximation; CI = Confidence Interval. *p < 0.001.

**Table 3:** Descriptive statistics and item-factor loadings of the five-factor Dutch CHANT model.

		Standardized Factor Loadings					
	Mean (*SD*)	Awareness		Concern	Motivation	Home behaviors	Work behaviors
**1. Awareness (BEW1):** earth warming and climate change	4.24 (0.84)	0.59					
**2. Awareness (BEW2):** greenhouse gases and climate change	4.34 (0.75)	0.67					
**3. Awareness (BEW3):** healthcare delivery and greenhouse gas emissions	3.44 (1.20)	0.60					
**4. Awareness (BEW4):** adverse health conditions and climate change	3.77 (1.06)	**0.84**					
**5. Awareness (BEW5):** vulnerable populations and climate change	3.80 (1.12)	**0.81**					
**6. Concern (BEZ1):** health impacts	3.61 (1.02)			**0.78**			
**7. Concern (BEZ2):** financial impacts	3.67 (1.03)			**0.72**			
**8. Concern (BEZ3):** impact on self and family	3.47 (0.99)			**0.72**			
**9. Concern (BEZ4):** impact on future generations	4.19 (0.97)			**0.83**			
**10. Concern (BEZ5):** impact on earth	4.05 (1.11)			**0.87**			
**11. Motivation (MOT1):** change to own nursing practice	4.12 (0.97)				**0.81**		
**12. Motivation (MOT2):** educate on climate change and health impacts	3.59 (1.04)				**0.85**		
**13. Motivation (MOT3):** prepare for health impacts at own workplace	3.69 (1.01)				**0.73**		
**14. Behavior at home (GED1_1):** use non-fossil fuel-based energy sources	3.48 (1.12)					0.33	
**15. Behavior at home (GED1_2):** conserve energy	4.07 (0.72)					0.64	
**16. Behavior at home (GED1_3):** use less gasoline	3.66 (0.96)					0.68	
**17. Behavior at home (GED1_4):** reduce waste	3.67 (0.89)					**0.76**	
**18. Behavior at home (GED1_5):** choose food with lower resource necessity	3.29 (0.99)					**0.79**	
**19. Behavior at work (GED2_1):** conserve energy	3.82 (0.79)						0.57
**20. Behavior at work (GED2_2):** commute to work (bike, walk, public transport)	3.34 (1.27)						0.61
**21. Behavior at work (GED2_3):** reduce waste	3.52 (0.93)						**0.75**
**22. Behavior at work (GED2_4):** ask leaders to reduce greenhouse gases	2.47 (1.18)						0.65
**McDonald's ω / Cronbach's α**		0.84/0.82		0.88/0.89	0.84/0.84	0.76/0.76	0.73/0.73

Note. All loadings are statistically significant, p < .001. Bolded loadings represent loadings over 0.70. Abbreviations: SD = Standard Deviation.

When compared with the original Climate and Health Tool, the Dutch version displayed slightly lower model fit indices, but the overall patterns of factor loadings remained highly comparable (e.g. item 10: concern - demonstrating a factor loading of 0.94 in the original and 0.87 in the translation) (Winquist et al., 2023). Likewise, internal consistency showed close alignment; for instance the *home behavior* factor demonstrated a Cronbach's α value of 0.75 for the original and 0.76 for the Dutch version (Winquist et al., 2023). In consultation with a statistical expert, we decided not to modify the current Dutch version of the instrument. Adjusting the model based solely on statistical output risks introducing model misspecification or overfitting (Brown and Moore, 2012), and may compromise content validity by altering items that are theoretically grounded (Sutharson et al., 2025). Such changes could ultimately weaken, rather than strengthen, the integrity of the instrument.

## 4 Discussion

The objective of this study was to establish a rigorous cross-cultural adaptation, translation and validation of the Climate and Health Tool into Dutch and to evaluate its psychometric properties. To ensure cultural and contextual relevance, the instrument was adapted for the Dutch context through careful translation, expert committee review, and pre-testing, resulting in minor textual revisions. Interviews revealed generally good comprehensibility, but highlighted ambiguities that required revisions to improve overall interpretability of the instrument for the Dutch context. The confirmatory factor analysis indicated that the Dutch 22-item, five-factor model demonstrated moderate fit according to commonly used cut-off criteria (Hu and Bentler, 1999; Schermelleh-Engel et al., 2003; Hooper et al., 2008; Brown, 2015; McNeish et al., 2018), with two out of four fit indices meeting the cited thresholds. Internal consistency analyses showed positive internal consistency all-round. When compared with the psychometric properties of the original Climate and Health Tool, the Dutch version displayed slightly lower fit indices, but similar patterns of factor loadings and internal consistency. In the interest of rigor, the slightly lower model fit observed in the Dutch version was explored statistically and in literature. The slightly lower model fit could be attributed to subtle cultural nuances, a presence of high modification indices (supplementary file V), or decisions made during the development of the original instrument (Schenk et al., 2019). However, the parallels in factor loading and internal consistency, along with the rigorous cultural adaptation and translation process, support the conceptual, semantic, experiential, and idiomatic equivalence and the stability of the translated instrument.

This study demonstrates several strengths. The translation and cultural adaptation of the instrument were conducted using a rigorous and systematic methodology, following the guidelines of Beaton et al. (2000). This systemic approach enhanced both the linguistic and conceptual, semantic, experiential, and idiomatic equivalence of the Dutch version. A second strength lies in the involvement of a broad, multidisciplinary expert committee, including a certified translator, nurse practitioners, lecturers, and researchers. This diversity ensured that the instrument was evaluated from multiple professional perspectives, strengthening its content validity. Additionally, the research team maintained continuous dialogue with the developer of the original instrument, which safeguarded fidelity and conceptual integrity throughout the adaptation process and supported consistent decision-making. The pre-testing phase further contributed to the study's robustness. By engaging a diverse group of nurses, ranging from nurse practitioners to students and lecturers, the usability, clarity, and relevance of the instrument were thoroughly assessed in a real-world context covering education and working practice. Finally, the psychometric analyses were based on a heterogeneous and sufficiently powered sample. The inclusion of nurses from various healthcare settings strengthens the generalizability of the findings within the nursing profession.

Some limitations are present in this study. First, findings from the factor analysis suggest that certain model refinement could be considered; however, the instrument was retained in its original form to ensure conceptual comparability with previous validations. As a result, some fit indices fall slightly below commonly cited thresholds (Hu and Bentler, 1999). Second, efforts were made to ensure neutral participant recruitment (e.g. neutral invitation to participate). However, factor means reflected positive scores across the instrument that were statistically significantly higher than the instrument's midpoint score. This could potentially reflect a self-selection bias and limit generalizability of the findings to nurses with lower levels of interest in climate change and health. At the same time, the characteristics of the sample (e.g. size and heterogeneity) limits this possibility. Finally, the study focused solely on nurses, while the instrument was designed for broader use among healthcare professionals (Rangel et al., 2024). Given that climate-related health impacts affects the entire healthcare sector, future research should focus on continued evaluation of the instrument's performance across multiple health professional groups to strengthen its psychometric validation. Additionally, subsequent research should emphasize model refinement to further enhance the instrument's validity and, by extension, its overall integrity. Future research should also focus on establishing a comprehensive baseline of awareness, concern, motivation, and behavior regarding climate change and health across multiple health professional groups, so that existing gaps are identified and focused interventions can be developed and aimed at addressing profession-specific needs and strengthening competencies. Moreover, this study may serve as a methodological reference for future cross-cultural adaptation research involving the Climate and Health Tool, offering guidance on research design and procedure.

As the first Dutch translation of the Climate and Health Tool, this instrument offers several valuable applications for Dutch nursing practice, education, and research. It enables researchers to systematically assess, monitor, and compare nurses’ awareness, concern, motivation, and behavior regarding climate-related health issues over time and context, providing a foundation for evaluating the effectiveness of interventions and identifying areas requiring additional support. In nursing education, the instrument can help programs identify gaps in climate-related competencies and guide targeted curriculum development. This aligns closely with emerging national frameworks such as the BN2030 Dutch nursing curriculum profile (Bouwes et al., 2023), which emphasizes sustainability and the evolving role of nurses in climate-resilient care. Within practice settings, the instrument can be used to assess training needs and ensure that nurses are equipped with the knowledge and skills required to respond to climate-sensitive health risks. Beyond assessment, the instrument can function as a catalyst for broader organizational and sector-wide initiatives, supporting the development of new policies, sustainability programs, and strategic actions aimed at strengthening climate mitigation and adaptation across healthcare. Because nurses serve as a highly trusted interface between health systems and society, they are uniquely positioned to lead and accelerate climate-related health initiatives. By providing a standardized and validated method to measure climate-related competencies, this instrument contributes to strengthening the capacity of Dutch healthcare systems to anticipate, respond to, and mitigate climate-related challenges.

## 5 Conclusion

This study introduces a reliable and validated Dutch instrument to assess nurses' awareness, concern, motivation, and behavior regarding climate change and health: the Dutch version of the Climate and Health Tool. The validated Dutch instrument enables researchers, educators, and healthcare organizations to systematically evaluate climate-related competencies of nurses and to identify areas were additional support, training, or curricular development is required. Overall, this instrument forms an important foundation for accelerating climate action within Dutch healthcare and supports efforts to better prepare health systems for current and emerging climate-related challenges.

## Declaration of generative AI use

During the preparation of this work the author(s) used Copilot in order to copyedit parts of the first draft, and support coding in Rstudio. After using this tool/service, the author(s) reviewed and edited the content as needed and take(s) full responsibility for the content of the published article.

## Funding

This research did not receive any specific grant from funding agencies in the public, commercial, or not-for-profit sectors.

## CRediT authorship contribution statement

**Rick R.A. Hendriks:** Writing – review & editing, Writing – original draft, Visualization, Project administration, Methodology, Investigation, Formal analysis, Data curation, Conceptualization. **Leontien van Wely:** Writing – review & editing, Supervision, Formal analysis, Conceptualization. **Yara Gutter:** Writing – original draft, Investigation, Formal analysis, Conceptualization. **Monique Chambon:** Writing – review & editing, Investigation. **Nicole Hunfeld:** Writing – review & editing, Investigation. **Kim J. Verhaegh:** Writing – review & editing, Supervision, Resources, Funding acquisition, Conceptualization.

## Declaration of competing interest

I have nothing to declare.

## Data Availability

A dataset is made available through DANS Data Stations:https://doi.org/10.17026/SS/FKKJUM

## Appendix Supplementary materials

Image, application 1

## References

[bib0001] Albrecht G.A. (2019).

[bib0002] Beaton D.E., Bombardier C., Guillemin F., Ferraz M.B. (2000). Guidelines for the process of Cross-Cultural adaptation of Self-Report measures. Spine.

[bib0003] Bouwes A., Broekman Ms., RN Dobber, R Eisenberg, R Hertog, Rutgers R. (2023). *Opleidingsprofiel BN2030*. Available from.

[bib0004] Brown T.A. (2015).

[bib0005] Brown T.A., Moore M.T. (2012). Confirmatory factor analysis. Handb. Struct. Equ. Model..

[bib0006] Byrne B.M. (1994). Testing for the factorial validity, replication, and invariance of a measuring instrument: a paradigmatic application based on the Maslach Burnout inventory. Multivar. Behav Res..

[bib0007] Cruchinho P., López-Franco M.D., Capelas M.L., Almeida S., Bennett P.M., Da Silva M.M., Teixeira G., Nunes E., Lucas P., Gaspar F. (2024). Translation, cross-cultural adaptation, and validation of measurement instruments: a practical guideline for novice researchers. J Multidiscip Heal..

[bib0008] Eitelwein O., Fricker R., Green A., Racloz V., Baid H., Bochkov M., Díaz Cely D., Gómez F.J., Moore J., Wautier T., Wechsler P. (2024). https://www3.weforum.org/docs/WEF_Quantifying_the_Impact_of_Climate_Change_on_Human_Health_2024.pdf.

[bib0009] Hooper D., Coughlan J., Mullen M. (2008). Structural equation modelling: guidelines for determining model fit. Electron. J. Bus. Res. Methods.

[bib0010] Hu L., Bentler P.M. (1999). Cutoff criteria for fit indexes in covariance structure analysis: conventional criteria versus new alternatives. Struct. Equ. Model. Multidiscip. J..

[bib0011] International Council of Nurses (2024). *Nurses, climate change and health* (Position statement). https://www.icn.ch/sites/default/files/202411/Nurses%20climate%20change%20health%20PS_EN.pdf.

[bib0012] Kline P. (1994).

[bib0013] Kotcher J., Maibach E., Miller J., Campbell E., Alqodmani L., Maiero M., Wyns A. (2021). Views of health professionals on climate change and health: a multinational survey study. Lancet Planet. Health.

[bib0014] Kyriazos T.A. (2018). Applied psychometrics: sample size and sample power considerations in factor analysis (EFA, CFA) and SEM in general. Psychology.

[bib0015] Lee J.J., Huang Y., Yan Y., Lui Y.W., Ye F. (2024). Integrating climate change and sustainability in nursing education. Nurse Educ. Today.

[bib0016] Levett-Jones T., Catling C., Cheer S., Fields L., Foster A., Maguire J., Mcintyre E., Oam T.M., Pich J., Pitt V., Whiteing N., Lokmic-Tomkins Z. (2025). Achieving consensus on the essential knowledge and skills needed by nursing students to promote planetary health and sustainable healthcare: a Delphi study. J. Adv. Nurs..

[bib0017] McDonald R.P. (1999).

[bib0018] McNeish D., An J., Hancock G.R. (2018). The thorny relation between measurement quality and fit index cutoffs in latent variable models. J. Pers. Assess..

[bib0019] Müller F., Skok J.I., Arnetz J.E., Bouthillier M.J., Holman H.T. (2024). Primary care clinicians’ attitude, knowledge, and willingness to address climate change in shared decision-making. J. Am. Board Fam. Med..

[bib0020] Planetary Health Alliance (2026). What is Planetary Health? - Planetary Health Alliance. https://planetaryhealthalliance.org/what-is-planetary-health/.

[bib0021] Quarsie, J., van de Pas, R., Fanoy, E., & van den Hazel, P. (2021). De impact van klimaatverandering op gezondheid in Nederland. Nederlands Tijdschrift voor Geneeskunde, 165, D6245. Available from: de impact van klimaatverandering op gezondheid in Nederland | NTVG [accessed Feb 09 2026]. 34523842

[bib0022] Rangel T., Johnson S.E., Joubert P., Timmerman R., Smith S., Springer G., Schenk E. (2025). Comparisons of healthcare personnel relating to awareness, concern, motivation, and behaviours of climate and health: a cross-sectional study. J. Adv. Nurs..

[bib0023] Rijksdienst voor Ondernemend Nederland. (n.d.). *Green Deals.* Available from: green Deals | RVO.Nl [accessed Jun 10 2026].

[bib0024] Ryan E.C., Dubrow R., Sherman J.D. (2020). Medical, nursing, and physician assistant student knowledge and attitudes toward climate change, pollution, and resource conservation in health care. BMC. Med. Educ..

[bib0025] Salvage J., White J. (2020). Our future is global: nursing leadership and global health. Rev Lat Am Enferm..

[bib0026] Santos O.P.D., Perruchoud É., Pereira F., Alves P., Verloo H. (2024). Measuring nurses’ knowledge and awareness of climate change and Climate-associated diseases: systematic review of existing instruments. Nurs. Rep..

[bib0027] Santos O.P.D., Alves P.J.P., Verloo H. (2025). The CHANT’s conceptual and psychometric validity in Switzerland: a descriptive three-round multicentre E-Delphi study. Nurs. Rep..

[bib0028] Schenk E.C., Cook C., Demorest S., Burduli E. (2019). CHANT: climate, Health, and nursing tool: item development and exploratory factor analysis. Annu. Rev. Nurs. Res..

[bib0029] Schenk E.C., Cook C., Demorest S., Burduli E. (2021). Climate, Health, and Nursing Tool (CHANT): initial survey results. Public Health Nurs..

[bib0030] Schermelleh-Engel K., Moosbrugger H., Müller H. (2003). Evaluating the fit of structural equation models: tests of significance and descriptive goodness-of-fit measures. Psychol. Arch..

[bib0031] Staatsen B., Hall E., Strak M., Betgen C., Limaheluw J., Mulder Y., van Bakel M., Kupper N., Deuning C., Couwenbergh C., den Broeder L. (2024).

[bib0032] Steenmeijer M.A., Rodrigues J.F., Zijp M.C., Waaijers-van der Loop S.L. (2022). The environmental impact of the Dutch health-care sector beyond climate change: an input–output analysis. Lancet Planet. Health.

[bib0033] Sutharson P., Maaskant J., Eskes A. (2025). The Dutch-translated cultural competence assessment scale for nurses: cross-cultural adaptation and validation. Int. J. Nurs. Stud. Adv..

[bib0034] Tavakol M., Dennick R. (2011). Making sense of Cronbach's alpha. Int. J. Med. Educ..

[bib0035] Wuebbles D.J., Fahey D.W., Hibbard K.A., Dokken D.J., Stewart B.C., Maycock T.K., USGCRP (2017). Climate Science Special Report: Fourth National Climate Assessment, Volume I.

[bib0036] Vandenberg S., Strus J.A., Chircop A., Egert A., Savard J. (2025). Planetary Health in Nursing: a scoping review. J. Adv. Nurs..

[bib0037] Whitmee S., Haines A., Beyrer C., Boltz F., Capon A.G., De Souza Dias B.F., Ezeh A., Frumkin H., Gong P., Head P., Horton R., Mace G.M., Marten R., Myers S.S., Nishtar S., Osofsky S.A., Pattanayak S.K., Pongsiri M.J., Romanelli C., Yach D. (2015). Safeguarding human health in the Anthropocene epoch: report of The Rockefeller Foundation–Lancet Commission on planetary health. Lancet.

[bib0038] Winquist A., Schenk E.C., Cook C., Demorest S., Burduli E. (2023). Climate, Health, and Nursing Tool (CHANT): a confirmatory factor analysis. Public Health Nurs..

[bib0039] World Health Organization (2020).

[bib0040] World Health Organization (2025).

