# Supplementary material for: The Dutch-translated Climate and Health Tool for nurses: cross-cultural adaptation and validation
Source: Int J Nurs Stud Adv. 2026 Jun 17;11:100603. doi: 10.1016/j.ijnsa.2026.100603 (PMC13320510; doi:10.1016/j.ijnsa.2026.100603)

**Supplementary file I.** *Original Climate, Health, and Nursing Tool.*

| Awareness: Please indicate your level of familiarity with the following evidence-based statements.    Awareness Scale:  1 = Not at all familiar  2 = Slightly familiar  3 = Somewhat familiar  4 = Moderately familiar  5 = Extremely familiar |
| --- |
| - The planet has warmed significantly since the 1850s, causing climate change. |
| - The warming which causes climate change is due in large part to human behaviors which add greenhouse gases (GHGs) to the atmosphere (such as use of gas and coal to create electricity and heat buildings, fuel for transportation, and modern agriculture) |
| - Health care delivery is responsible for approximately 8.5% of total greenhouse gas emissions in the US that contribute to warming. |
| - Climate change increases the likelihood of adverse health conditions such as heat stroke, asthma exacerbation, Lyme disease, and others. |
| - Vulnerable populations such as the very young or old, and other at-risk groups (people living with homelessness or poverty, people of color, etc.) experience more adverse health impacts from climate change. |
| Concern: How concerned are you about the following, as they relate to climate change?    Concern Scale:  1 = Not at all  2 = Slightly  3 = Somewhat  4 = Moderately  5 = Extremely |
| - Health impacts |
| - Financial impacts (rebuilding after storms or fires, health costs, etc.) |
| - Overall impacts on you, your family, or someone you know today. |
| - Overall impacts on future generations |
| - Changes to the planet (other species, forests, oceans, etc.) |
| Motivation: Health care contributes approximately 8.5% of Greenhouse Gases (GHGs) in the US. Please indicate how true the following statements are *for you.*    Motivation Scale:  1 = Very untrue for me  2 = Somewhat untrue for me  3 =Neutral  4 = True for me  5 = Very true for me  Not applicable |
| - I want to change my practice to reduce GHG contributions. |
| - I want to teach patients/clients/community members about how climate change impacts health. |
| - I want to prepare for health impacts of climate change at my workplace. |
| Behavior at home: How often do you perform the following behaviors at *home*:    Behavior scale:  1 = Never  2 = Rarely  3 = Sometimes  4 = Often  5 = Always |
| - Use non-fossil fuel-based energy sources (such as purchase wind or solar energy, geo-thermal, buy energy offsets, etc.) |
| - Conserve energy (such as use energy efficient appliances, keep moderate temperature settings, turn off lights and electronics, etc.) |
| - Use less gasoline (drive fuel-efficient vehicles, reduce unnecessary trips, bike-walk, etc.) |
| - Reduce waste (buy less, reuse more, recycle and compost more) |
| - Choose foods that require fewer resources to grow/produce (local, seasonal, fewer animal products, less packaging) |
| Behavior at work: How often do you do the following behaviors at work: (If you do not work or volunteer in a professional setting, please skip this question)  Behavior scale:  1 = Never  2 = Rarely  3 = Sometimes  4 = Often  5 = Always |
| - Conserve energy (such as turn off lights and electronics, etc.) |
| - Commute to work using active (bike, walk), shared, or public transportation. |
| - Reduce waste (plastic, paper, linen, clinical supplies, etc.) |
| - Ask leaders at your workplace to support policies, products and/or processes that create fewer greenhouse gases (GHGs) |

**Supplementary file II.** *Translated* *Climate, Health, and Nursing Tool in Dutch.*

| Bewustzijn: Geef aan in hoeverre u bekend bent met de volgende wetenschappelijk onderbouwde uitspraken.    Bewustzijn schaal:  1 = Helemaal niet bekend  2 = Een klein beetje bekend  3 = Enigszins bekend  4 = Redelijk bekend  5 = Heel erg bekend |
| --- |
| - De aarde is sinds de jaren 1850 aanzienlijk opgewarmd, wat klimaatverandering tot gevolg heeft. |
| - De opwarming die leidt tot klimaatverandering is voor een groot deel te wijten aan menselijk gedrag, waarbij broeikasgassen in de atmosfeer worden uitgestoten (zoals gebruik van gas en steenkool om elektriciteit op te wekken en gebouwen te verwarmen, en gebruik van brandstof voor vervoer en moderne landbouw) |
| - De gezondheidszorg is verantwoordelijk voor ongeveer 7% van de totale uitstoot van CO2 in Nederland, die bijdraagt aan de opwarming van de aarde. |
| - Klimaatverandering verhoogt de kans op gezondheidsproblemen zoals hitteberoerte, verergering van astma, de ziekte van Lyme en andere. |
| - Kwetsbare bevolkingsgroepen zoals zeer jonge of oude mensen en andere risicogroepen (lage sociaaleconomische positie, chronisch zieken) ervaren meer gezondheidsproblemen vanwege klimaatverandering. |
| Bezorgdheid: Hoe bezorgd bent u over het volgende, in relatie tot klimaatverandering?    Bezorgdheid Schaal:  1 = Helemaal niet  2 = Een klein beetje  3 = Enigszins  4 = Redelijk  5 = Heel erg |
| - Gevolgen voor de gezondheid. |
| - Financiële gevolgen (kosten voor wederopbouw na stormen of branden, zorgkosten, etc.) |
| - Gevolgen voor uzelf, uw familie of een bekende. |
| - Gevolgen voor toekomstige generaties. |
| - Veranderingen aan de aarde (bijv. andere plant- en diersoorten, bossen, oceanen, etc.) |
| Motivatie: Zoals eerder gemeld is de gezondheidszorg verantwoordelijk voor ongeveer 7% van de totale uitstoot van CO2 in Nederland. Geef aan in hoeverre de volgende uitspraken op u van toepassing zijn:    Motivatie schaal:  1 = Zeer onwaar voor mij  2 = Enigszins onwaar voor mij  3 = Neutraal  4 = Waar voor mij  5 = Zeer waar voor mij  Niet van toepassing |
| - Ik wil mijn werkwijze veranderen om de uitstoot van broeikasgassen te verminderen. |
| - Ik wil patiënten/cliënten/mensen in de gemeenschap leren hoe klimaatverandering de gezondheid beïnvloedt. |
| - Ik wil mijn werkplek voorbereiden op de gezondheidsgevolgen van klimaatverandering (*bijv. voorbereiden op onverwachte toename van vraag naar gezondheidszorg of verminderde beschikbaarheid van personeel tijdens extreme gebeurtenissen, etc.*) |
| Gedrag thuis: Hoe vaak vertoont u het volgende gedrag *thuis*?    Gedrag schaal:  1 = Nooit  2 = Zelden  3 = Soms  4 = Vaak  5 = Altijd |
| - Energiebronnen gebruiken die niet op fossiele brandstoffen zijn gebaseerd (*zoals het inkopen van wind- of zonne-energie, aardwarmte, etc*.) |
| - Energie besparen (*zoals het gebruik van energiezuinige apparaten, de thermostaat lager instellen, lichten en elektronica uitschakelen, etc.)* |
| - Minder brandstof gebruiken (*rijden in energiezuinige voertuigen, verminderen van onnodige ritten, fietsen, lopen etc.).* |
| - Afval verminderen (*minder kopen, meer hergebruiken, recyclen en meer composteren*) |
| - Voedingsmiddelen kiezen die minder grondstoffen nodig hebben om te groeien/produceren (*lokaal, seizoensgebonden, minder dierlijke producten, minder verpakkingsmateriaal*). |
| Gedrag op werk: Hoe vaak vertoont u het volgende gedrag op het werk? (als u niet werkt óf geen vrijwilligerswerk doet, sla deze vraag over).  Gedrag schaal:  1 = Nooit  2 = Zelden  3 = Soms  4 = Vaak  5 = Altijd |
| - Energie besparen (*zoals lichten en elektronica uitschakelen, etc.)* |
| - Woon-werkverkeer via actief vervoer (*fietsen, lopen*), gedeeld (*carpoolen*) of openbaar vervoer. |
| - Afval verminderen (*plastic, papier, linnen, klinische benodigdheden, etc.)* |
| - Leidinggevenden op uw werkplek vragen om beleid, producten en/of processen te ondersteunen die minder broeikasgassen uitstoten. |

**Supplementary file III.** *Expert committee table containing all versions of the CHANT to consolidate a pre-final version.*

**Awareness**

| English [Original] | Dutch [Translation 1] | Dutch [Translation 2] | Dutch [Synthesis] | Dutch [Expert Committee consolidation] | English [Back translation 1] | English [Back translation 2] |
| --- | --- | --- | --- | --- | --- | --- |
| **Awareness 1:** Please indicate your level of familiarity with the following evidence-based statements.    **Awareness Scale:**  1 = Not at all familiar  2 = Slightly familiar  3 = Somewhat familiar  4 = Moderately familiar  5 = Extremely familiar | **Bewustzijn 1:** Geef aan hoe bekend je bent met de volgende op bewijs gebaseerde uitspraken.    **Bewustzijn schaal:**  1 = Helemaal niet bekend  2 = Een beetje bekend  3 = Enigszins bekend  4 = Redelijk bekend  5 = Uiterst bekend | **Bewustzijn 1:** Geef alstublieft aan in hoeverre u bekend bent met de volgende wetenschappelijk onderbouwde stellingen.    **Bewustzijns Schaal:**  1 = Helemaal niet bekend  2 = Een heel klein beetje bekend  3 = Enigszins bekend  4 = Redelijk bekend  5 = Heel erg bekend | **Bewustzijn 1:** Geef aan in hoeverre u bekend bent met de volgende wetenschappelijk onderbouwde uitspraken.    **Bewustzijn schaal**:  1 = Helemaal niet bekend  2 = Een klein beetje bekend  3 = Enigszins bekend  4 = Redelijk bekend  5 = Heel erg bekend | **Bewustzijn 1:** Geef aan in hoeverre u bekend bent met de volgende wetenschappelijk onderbouwde uitspraken.    **Bewustzijn schaal**:  1 = Helemaal niet bekend  2 = Een klein beetje bekend  3 = Enigszins bekend  4 = Redelijk bekend  5 = Heel erg bekend | **Awareness 1:** Please indicate the extent to which you are familiar with the following scientifically based statements.  **Awareness scale**  1 = Not known at all  2 = A little known  3 = Somewhat familiar  4 = Fairly well known  5 = Very well known | **Awareness 1:** Please, rate your level of awareness of the following scientifically based statements.  **Awareness scale:**  1 = Not aware at all  2 = Slightly aware  3 = Moderately aware  4 = Reasonably aware  5 = Very much aware |
| - The planet has warmed significantly since the 1850s, causing climate change. | - De aarde is sinds de jaren 1850 aanzienlijk opgewarmd, wat klimaatverandering veroorzaakt. | - De planeet is aantoonbaar opgewarmd sinds 1850, wat klimaatverandering tot gevolg heeft. | - De planeet is sinds de jaren 1850 aanzienlijk opgewarmd, wat klimaatverandering tot gevolg heeft. | - De planeet is sinds de jaren 1850 aanzienlijk opgewarmd, wat klimaatverandering tot gevolg heeft. | - The planet has warmed significantly since the 1850s, causing climate change. | - The planet has warmed considerably since the 1850s, resulting in a climate change. |
| - The warming which causes climate change is due in large part to human behaviors which add greenhouse gases (GHGs) to the atmosphere (such as use of gas and coal to create electricity and heat buildings, fuel for transportation, and modern agriculture) | - De opwarming die klimaatverandering veroorzaakt is voor een groot deel te wijten aan menselijk gedrag dat broeikasgassen (BKG's) aan de atmosfeer toevoegt (zoals het gebruik van gas en steenkool om elektriciteit op te wekken en gebouwen te verwarmen, brandstof voor transport en moderne landbouw) | - De opwarming die leidt tot klimaatverandering, is voornamelijk te wijten aan menselijk gedrag waarbij broeikasgassen (BKG'n) in de atmosfeer vrijkomen (zoals gebruik van gas en steenkool om elektriciteit op te wekken en gebouwen te verwarmen, brandstof voor vervoer, en moderne landbouw) | - De opwarming, die leidt tot klimaatverandering, is voor een groot deel te wijten aan menselijk gedrag waarbij broeikasgassen in de atmosfeer vrijkomen (zoals gebruik van gas en steenkool om elektriciteit op te wekken en gebouwen te verwarmen, brandstof voor vervoer en moderne landbouw) | - De opwarming die leidt tot klimaatverandering is voor een groot deel te wijten aan menselijk gedrag waarbij broeikasgassen in de atmosfeer worden uitgestoten (zoals gebruik van gas en steenkool om elektriciteit op te wekken en gebouwen te verwarmen, en gebruik van brandstof voor vervoer en moderne landbouw) | - Warming, which leads to climate change, is largely due to the human behaviour that releases greenhouse gases into the atmosphere (such as use of gas and coal to generate electricity and heat buildings, fuel for transportation and modern agriculture). | - (Global) warming, resulting in climate change, is partly due to human behavior that causes the emission of greenhouse gases (e.g., the use of gas and coal to produce electricity and to heat buildings, fuel for transportation, and modern agriculture). |
| - Health care delivery is responsible for approximately 8.5% of total greenhouse gas emissions in the US that contribute to warming. | - De gezondheidszorg is verantwoordelijk voor ongeveer 7% van de totale uitstoot van CO2 in Nederland, die bijdragen aan de opwarming. | - Het leveren van zorg is verantwoordelijk voor ongeveer 8,5% van de totale uitstoot van broeikasgas in de VS die bijdraagt aan opwarming van de aarde. | - De gezondheidszorg is verantwoordelijk voor ongeveer 7% van de totale uitstoot van CO2 in Nederland, die bijdraagt aan de opwarming van de planeet. | - De gezondheidszorg is verantwoordelijk voor ongeveer 7% van de totale uitstoot van CO2 in Nederland, die bijdraagt aan de opwarming van de aarde. | - Healthcare accounts for about 7 % of the Dutch total CO_2_ emissions, which contributes to global warming. | - Health care is responsible for around 7% of the total carbon dioxide emission in the Netherlands that contributes to global warming. |
| - Climate change increases the likelihood of adverse health conditions such as heat stroke, asthma exacerbation, Lyme disease, and others. | - Klimaatverandering verhoogt de kans op ongunstige gezondheidsproblemen zoals een zonnesteek, verergering van astma, de ziekte van Lyme en andere. | - Klimaatverandering vergroot de kans op nadelige gezondheidssituaties, zoals hitteberoerte, verergering van astma, de ziekte van Lyme, en andere. | - Klimaatverandering verhoogt de kans op nadelige gezondheidssituaties zoals hitteberoerte, verergering van astma, de ziekte van Lyme en andere. | - Klimaatverandering verhoogt de kans op nadelige gezondheidssituaties gezondheidsproblemen zoals hitteberoerte, verergering van astma, de ziekte van Lyme en andere. | - Climate change increases the risk of adverse health conditions such as heat stroke, exacerbation of asthma, Lyme disease and others. | - Climate change increases the risk of adverse health conditions like heatstroke, asthma exacerbation, Lyme disease and other. |
| - Vulnerable populations such as the very young or old, and other at-risk groups (people living with homelessness or poverty, people of color, etc.) experience more adverse health impacts from climate change. | - Kwetsbare bevolkingsgroepen zoals zeer jonge of oude mensen en andere risicogroepen (dakloze mensen of mensen die in armoede leven) ervaren meer nadelige gezondheidseffecten van klimaatverandering. | - Kwetsbare bevolkingsgroepen, zoals de hele jonge of oude mensen, en andere risicogroepen (mensen zonder huis of levend in armoede, mensen van kleur, etc.) ervaren meer nadelige gezondheidseffecten vanwege klimaatverandering. | - Kwetsbare bevolkingsgroepen zoals zeer jonge of oude mensen en andere risicogroepen (mensen zonder huis of mensen die in armoede leven) ervaren meer nadelige gezondheidseffecten vanwege klimaatverandering. | - Kwetsbare bevolkingsgroepen zoals zeer jonge of oude mensen en andere risicogroepen (lage sociaal economische positie, chronisch zieken) ervaren meer gezondheidsproblemen vanwege klimaatverandering. | - Vulnerable populations such as the very young or old and other high-risk groups (people without homes of those living in poverty) experience more adverse health effects because of climate change. | - Vulnerable population groups like very young or old people and other at-risk populations (people experiencing homelessness or living in poverty) experience more adverse health outcomes due to climate change. |
| **Awareness 2:** I have heard about climate change from these sources (check all that apply). | **Bewustzijn 2:** Ik heb gehoord over klimaatverandering via deze bronnen (vink alles aan wat van toepassing is). | **Bewustzijn 2:** Ik heb via deze bronnen gehoord over klimaatverandering (kruis alles aan wat van toepassing is). | **Bewustzijn 2:** Ik heb via deze bronnen gehoord over klimaatverandering (vink alles aan wat van toepassing is). | **Bewustzijn 2:** Ik heb via deze bronnen gehoord over klimaatverandering (selecteer alles wat van toepassing is). | **Awareness 2:** I have heard about climate change through these sources (tick all that apply): | **Awareness 2:** I heard about climate change from these sources (tick all that apply). |
| - Print media | - Gedrukte media | - Gedrukte media | - Gedrukte media | - Gedrukte media | - Print media. | - Printed media. |
| - TV News | - TV-Nieuws | - Nieuws op televisie | - Nieuws op TV | - Nieuws op TV | - News on TV. | - The news on TV. |
| - Social Media | - Sociale Media | - Social Media | - Social Media | - Social Media | - Social media. | - Social Media. |
| - Internet | - Internet | - Internet | - Internet | - Internet | - Internet. | - Internet. |
| - Professional courses | - Professionele cursussen | - Vakinhoudelijke cursussen | - Vakinhoudelijke cursussen | - Vakinhoudelijke cursussen | - Professional courses. | - Professional courses. |
| - Professional Organizations | - Professionele organisaties | - Beroepsverenigingen | - Organisaties | - Organisaties/Beroepsverenigingen | - Organizations. | - Organisations. |
| - Friends and/or family | - Vrienden en/of familie | - Vrienden en/of familie | - Vrienden en/of familie | - Vrienden en/of familie | - Friends and/ or family. | - Friends and/or family. |
| - Other____________ | - Anders_____________ | - Anders____________ | - Anders_____________ | - Anders_____________ | - Otherwise: | - Other …. |
| - I haven't heard of climate change | - Ik heb niet gehoord van klimaatverandering | - Ik heb niet over klimaatverandering gehoord | - Ik heb niet over klimaatverandering gehoord | - Ik heb niet over klimaatverandering gehoord | - I haven't heard about climate change. | - I have not heard about climate change. |

**Experience**

| English [Original] | Dutch [Translation 1] | Dutch [Translation 2] | Dutch [Synthesis] | Dutch [Expert Committee consolidation] | English [Back translation 1] | English [Back translation 2] |
| --- | --- | --- | --- | --- | --- | --- |
| **Experience 1:** How often have you noticed the following climate-related **weather events** in your area?    **Experience Scale:**  1 = Never  2 = Rarely  3 = Occasionally  4 = Frequently  5 = Very frequently | **Ervaring 1:** Hoe vaak heb je de volgende klimaat gerelateerde **weeromstandigheden** in jouw omgeving opgemerkt?    **Ervaring schaal:**  1 = Nooit  2 = Zelden  3 = Af en toe  4 = Vaak  5 = Zeer vaak | **Ervaring 1:** Hoe vaak heeft u de volgende klimaat-gerelateerde **weersomstandigheden**opgemerkt in uw omgeving?    **Ervarings Scaal:**  1 = Nooit  2 = Zelden  3 = Af en toe  4 = Regelmatig  5 = Zeer regelmatig | **Ervaring 1:** Hoe vaak heeft u de volgende klimaat-gerelateerde **weersomstandigheden** in uw omgeving opgemerkt?    **Ervaringsschaal**:  1 = Nooit  2 = Zelden  3 = Af en toe  4 = Vaak  5 = Zeer vaak | **Ervaring 1:** Hoe vaak heeft u de volgende klimaat-gerelateerde **weersomstandigheden** in uw omgeving opgemerkt?    **Ervaringsschaal**:  1 = Nooit  2 = Zelden  3 = Af en toe  4 = Vaak  5 = Zeer vaak | **Experience 1:** How often have you noticed the following climate-related **weather conditions** in your area?    **Experience scale:**  1 = Never  2 = Rarely  3 = Occasionally  4 = Often  5 = Very often | **Experience 1.** How often did you notice the following climate-related **weather conditions***?*    **Experience scale**:  1 = Never  2 = Seldom  3 = Occasionally  4 = Often  5 = Very often |
| - Extreme heat | - Extreme hitte | - Extreme hitte | - Extreme hitte | - Extreme hitte | - Extreme heat | - Extreme heat |
| - Heavy precipitation | - Zware regen | - Zware neerslag | - Zware neerslag | - Zware neerslag | - Heavy precipitation | - Heavy rain |
| - Droughts | - Droogte | - Droogte | - Droogte | - Droogte | - Drought | - Dryness |
| - Flooding | - Overstromingen | - Overstroming | - Overstromingen | - Overstromingen | - Floods | - Floods |
| - Hurricanes and storm surges | - Harde wind | - Orkanen en vloedgolven | - Orkanen en vloedgolven | - Orkanen Extreme storm, zware windstoten, of valwinden, en stormvloed | - Hurricanes and tidal waves | - Hurricanes and tidal waves |
| - Wildfires | - Bosbranden | - Natuurbranden | - Natuurbranden | - Natuurbranden | - Wild fires | - Wildfires |
| **Experience 2:** The Centers for Disease Control identify several health conditions that are worsened by climate change. For each group, **how often** are you seeing these conditions? | **Ervaring 2:** Het RIVM heeft verschillende gezondheidsproblemen geïdentificeerd die verergerd worden door klimaatverandering. Hoe vaak zie je deze aandoeningen voor elke groep? | **Ervaring 2:** Het Amerikaanse instituut CDC signaleert een aantal gezondheidssituaties die door klimaatverandering verergerd worden. Hoe vaak ziet u deze situaties, binnen iedere groep? | **Ervaring 2:** Het RIVM identificeert verschillende gezondheidssituaties die door klimaatverandering verergerd worden. **Hoe vaak** neemt u deze situaties waar voor iedere groep? | **Ervaring 2:** Het RIVM signaleert verschillende gezondheidsproblemen die door klimaatverandering verergerd worden. **Hoe vaak** ziet u deze gezondheidsproblemen voor iedere groep? | **Experience 2:** The National Institute for Public Health and the Environment (RIVM) identifies several health situations exacerbated by climate change**. How often** do you observe these situations for each group? | **Experience 2:** RIVM identifies several health conditions that worsen as a result of climate change. For each group, **how often** do you observe these conditions? |
| 2a: Patients/Clients: | 2a: Patiënten/Cliënten | 2a. Patiënten/cliënten: | 2a: Patiënten/Cliënten: | 2a: Patiënten/Cliënten: | 2a: Patients/ Clients: | 2a: Patients/Clients: |
| - Respiratory problems, such as asthma, allergies, or worsening COPD. | - Ademhalingsproblemen, zoals astma, allergieën of verergerende COPD. | - Ademhalingsproblemen, zoals astma, allergieën, of verergerende COPD. | - Ademhalingsproblemen, zoals astma, allergieën of verergerende COPD. | - Ademhalingsproblemen, zoals astma, allergieën of verergerende COPD. | - Respiratory problems such as asthma, allergies or worsening COPD. | - Respiratory problems, like asthma, allergies or exacerbation of COPD. |
| - Vector-borne diseases, such as Lyme disease, West Nile virus, Rocky Mountain Spotted Fever. | - Overdraagbare ziektes zoals malaria, ziekte van Lyme en het West-Nijlvirus. | - Vector-borne ziekten, zoals de ziekte van Lyme, het West-Nijl virus, Rocky Mountain gevlekte koorts. | - Vector-overgedragen ziekten zoals de ziekte van Lyme, West-Nijlvirus en Dengue. | - Vector-overgedragen ziekten zoals de ziekte van Lyme, West-Nijlvirus en Dengue. | - Vector-borne diseases such as Lyme disease, West Nile virus and Dengue. | - Vector-borne diseases, like Lyme disease, West Nile disease and dengue. |
| - Extreme heat illness | - Extreme hitte ziekte | - Extreme hitte ziekte. | - Extreme hitte ziekte. | - Hitte ziekte. | - Extreme heat illness. | - Extreme heat illness |
| - Physical trauma related to severe storms or fires. | - Fysieke trauma gerelateerd aan zware stormen of branden. | - Lichamelijk trauma gerelateerd aan zware stormen of branden. | - Fysieke trauma gerelateerd aan zware stormen of branden. | - Lichamelijk letsel door zware stormen of branden. | - Physical trauma related to severe storms or fires. | - Physical trauma related to heavy storms and fires. |
| - Mental health issues, such as depression, stress, anxiety, and/or trauma. | - Mentale gezondheidsproblemen, zoals depressie, stress, angst en/of trauma. | - Mentale gezondheidsproblemen, zoals depressie, stress, angst, en/of trauma. | - Mentale gezondheidsproblemen, zoals depressie, stress, angst en/of trauma. | - Mentale gezondheidsproblemen, zoals depressie, stress, angst en/of trauma. | - Mental health problems, such as depression, stress, anxiety and/ or trauma. | - Mental health problems, like depression, stress, anxiety and/or trauma. |
| 2b: Self/people I Know    … [see 2a.] | 2b: Zelf/mensen die ik ken    … [zie bij 2a.] | 2b. Mijzelf/mensen die ik ken:  … [zie bij 2a.] | 2b: Mijzelf/mensen die ik ken:  … [zie bij 2a.] | 2b: Mijzelf/mensen die ik ken:  … [zie bij 2a.] | 2b: Myself/ people I know:    … [see 2a.] | 2b: Myself/people who I know:  … [see 2a.] |
| 2c: People I read or hear about, but don’t know personally.    … [see 2a.] | 2c: Mensen over wie ik lees of hoor, maar niet persoonlijk ken.    … [zie bij 2a.] | 2c. Mensen waar ik over lees of hoor, maar niet persoonlijk ken.    … [zie bij 2a.] | 2c: Mensen over wie ik lees of hoor, maar niet persoonlijk ken.    … [zie bij 2a.] | 2c: Mensen over wie ik lees of hoor, maar niet persoonlijk ken.    … [zie bij 2a.] | 2c: people about whom I read or hear but do not know personally:    … [see 2a.] | 2c: People whom I read or hear about, but do not know personally:    … [see 2a.] |

**Concern**

| English [Original] | Dutch [Translation 1] | Dutch [Translation 2] | Dutch [Synthesis] | Dutch [Expert Committee consolidation] | English [Back translation 1] | English [Back translation 2] |
| --- | --- | --- | --- | --- | --- | --- |
| **Concern 1:** How **concerned** are you about the following, as they relate to climate change?    **Concern Scale:**  1 = Not at all  2 = Slightly  3 = Somewhat  4 = Moderately  5 = Extremely | **Zorg 1:** Hoe **bezorgd**ben je over het volgende, in relatie tot klimaatverandering?      **Zorg schaal:**   - Helemaal niet - Een beetje - Enigszins - Redelijk - Zeer | **Zorgen 1:** Hoe **bezorgd** bent u over de volgende zaken, wanneer die in verband worden gebracht met klimaatverandering?  **Zorgen Schaal:**  1 = Helemaal niet  2 = Een heel klein beetje  3 = Enigszins  4 = Redelijk  5 = Heel erg | **Bezorgdheid 1:** Hoe **bezorgd** bent u over het volgende, in relatie tot klimaatverandering?    **Zorgen Schaal**:  1 = Helemaal niet  2 = Een klein beetje  3 = Enigszins  4 = Redelijk  5 = Heel erg | **Bezorgdheid 1:** Hoe **bezorgd** bent u over het volgende, in relatie tot klimaatverandering?    **Zorgen Schaal**:  1 = Helemaal niet  2 = Een klein beetje  3 = Enigszins  4 = Redelijk  5 = Heel erg | **Concern 1:** How **concerned** are you about the following, in relation to climate change?    **Concern scale:**  1 = Not at all  2 = A little bit  3 = Somewhat  4 = Fair  5 = Very much | **Worry 1:** Concerning climate change, how **worried**are you about the following?    **Worry scale**:  1 = Not at all  2 = Slightly  3 = Moderately  4 = Fairly  5 = Very much |
| - Health impacts | - Gezondheidseffecten | - Impact op gezondheid | - Gevolgen voor de gezondheid. | - Gevolgen voor de gezondheid. | - Health implications | - Health impact |
| - Financial impacts (rebuilding after storms or fires, health costs, etc.) | - Financiële gevolgen (kosten voor wederopbouw na stormen of brand, gezondheidskosten etc.) | - Financiële impact (wederopbouw na stormen of branden, zorgkosten, etc) | - Financiële gevolgen (kosten voor wederopbouw na stormen of branden, zorgkosten, etc.) | - Financiële gevolgen (kosten voor wederopbouw na stormen of branden, zorgkosten, etc.) | - Financial implications (costs of rebuilding after storms or fires, healthcare costs etc.). | - Financial impact (cost for rebuilding after storms or fires, healthcare cost, et cetera). |
| - Overall impacts on you, your family, or someone you know today. | - Algemene gevolgen voor jou, je familie of iemand die je vandaag kent | - Algemene impact op uzelf, uw familie, of iemand die u vandaag ontmoet. | - Algemene gevolgen voor uzelf, uw familie of een bekende. | - Gevolgen voor uzelf, uw familie of een bekende. | - General implications for yourself, your family or an acquaintance. | - General consequences for yourself, your family or an acquaintance. |
| - Overall impacts on future generations | - Algemene gevolgen voor toekomstige generaties | - Algemene impact op toekomstige generaties | - Algemene gevolgen voor toekomstige generaties. | - Gevolgen voor toekomstige generaties. | - General implications for future generations | - General consequences for future generations. |
| - Changes to the planet (other species, forests, oceans, etc.) | - Veranderingen aan de planeet (andere soorten, bossen, oceanen, enz) | - Veranderingen op de planeet (andere soorten, bossen, oceanen, etc.) | - Veranderingen aan de planeet (andere soorten*, bossen, oceanen, etc.) | - Veranderingen aan de aarde (bijv. andereplant- en diersoorten*, bossen, oceanen, etc.) | - Changes to the planet (other species, forests, oceans etc.). | - Changes to the planet (different species, woods, oceans, et cetera). |

**Optimism**

| English [Original] | Dutch [Translation 1] | Dutch [Translation 2] | Dutch [Synthesis] | Dutch [Expert Committee consolidation] | English [Back translation 1] | English [Back translation 2] |
| --- | --- | --- | --- | --- | --- | --- |
| **Optimism 1:** How **optimistic** are you that humans will:    **Optimism Scale:**  1 = Not at all  2 = Slightly  3 = Somewhat  4 = Moderately  5 = Extremely | **Optimisme 1:** Hoe **optimistisch** ben je dat mensen:    **Optimisme schaal:**  1 = Helemaal niet  2 = Een beetje  3 = Enigszins  4 = Redelijk  5 = Zeer | **Optimisme 1:** Hoe **optimistisch** bent u dat mensen:    **Optimisme Schaal:**  1 = Helemaal niet  2 = Een heel klein beetje  3 = Enigszins  4 = Redelijk  5 = Heel erg | **Optimisme 1:** Hoe **optimistisch** bent u dat mensen:    **Optimisme schaal:**  1 = Helemaal niet  2 = Een klein beetje  3 = Enigszins  4 = Redelijk  5 = Heel erg | **Optimisme 1:** Hoe **optimistisch** bent u dat mensen:    **Optimisme schaal:**  1 = Helemaal niet  2 = Een klein beetje  3 = Enigszins  4 = Redelijk  5 = Heel erg | **Optimism 1:** How **optimistic** are you that people:    **Optimism scale:**  1 = Not at all  2 = A little bit  3 = Somewhat  4 = Fair  5 = Very much | **Optimism 1:** How **optimistic** are you that people:    **Optimism scale**:  1 = Not at all  2 = Slightly  3 = Moderately  4 = Fairly  5 = Very much |
| - Adequately ***prepare*** for the impacts of climate change? | - Adequaat zullen voorbereiden op de gevolgen van klimaatverandering? | - Zich voldoende zullen **voorbereiden**op de effecten door klimaatverandering | - Zich voldoende zullen **voorbereiden** op de gevolgen van klimaatverandering? | - Zich voldoende zullen **voorbereiden** op de gevolgen van klimaatverandering? | - Will they adequately **prepare** for the effects of climate change? | - Will **prepare** themselves for the consequences of climate change? |
| - ***Prevent*** further climate change? | - Verdere klimaatverandering zullen voorkomen? | - Verdere klimaatverandering zullen **voorkomen**? | - Verdere klimaatverandering zullen **voorkomen**? | - Verdere klimaatverandering zullen **voorkomen**? | - Further climate change will **occur**? | - Will **prevent** further climate change? |

**Motivation**

| English [Original] | Dutch [Translation 1] | Dutch [Translation 2] | Dutch [Synthesis] | Dutch [Expert Committee consolidation] | English [Back translation 1] | English [Back translation 2] |
| --- | --- | --- | --- | --- | --- | --- |
| **Motivation 1:** Health care contributes approximately 8.5% of Greenhouse Gases (GHGs) in the US. Please indicate how true the following statements are ***for you.***      **Motivation Scale:**  1 = Very untrue for me  2 = Somewhat untrue for me  3 =Neutral  4 = True for me  5 = Very true for me  Not applicable | **Motivatie 1:** De gezondheidszorg is verantwoordelijk voor ongeveer 7% van de totale uitstoot van CO2 in Nederland. Geef aan in hoeverre de volgende uitspraken waar zijn **voor jou**.    **Motivatie schaal:**  1 = zeer onwaar voor mij  2 = enigszins onwaar voor mij  3 = neutraal  4 = waar voor mij  5 = zeer waar voor mij  Niet van toepassing | **Motivatie 1:** De gezondheidszorg draagt bijna 8,5% bij aan de broeikasgassen in de VS. Geef alstublieft aan in hoeverre de volgende stellingen **voor u** gelden:      **Motivatie Schaal:**  1 = Geldt helemaal niet voor mij  2= Geldt niet voor mij  3 = Neutraal  4 = Geldt voor mij  5 = Geldt helemaal voor mij  Niet van toepassing | **Motivatie 1:** De gezondheidszorg is verantwoordelijk voor ongeveer 7% van de totale uitstoot van CO2 in Nederland. Geef aan in hoeverre de volgende uitspraken waar zijn **voor u**:    **Motivatie schaal**:  1 = Zeer onwaar voor mij  2 = Enigszins onwaar voor mij  3 = Neutraal  4 = Waar voor mij  5 = Zeer waar voor mij  Niet van toepassing | **Motivatie 1:** De gezondheidszorg is verantwoordelijk voor ongeveer 7% van de totale uitstoot van CO2 in Nederland. Geef aan in hoeverre de volgende uitspraken **op u** van toepassing zijn:    **Motivatie schaal**:  1 = Zeer onwaar voor mij  2 = Enigszins onwaar voor mij  3 = Neutraal  4 = Waar voor mij  5 = Zeer waar voor mij  Niet van toepassing | **Motivation 1:** Healthcare accounts for about 7% of total CO^2^ emissions in the Netherlands. Please indicate the extent to which the following statements are true **for you:**    **Motivation scale:**  1 = Very untrue for me  2 = Somewhat untrue for me  3 = Neutral  4 = True for me  5 = Very true for me  Not applicable | **Motivation 1:** Health care is responsible for about 7% of total C0_2_ emissions in the Netherlands. Please, indicate to what extent the following statements apply **to you**:      **Motivation scale**:  1 = Very untrue for me  2 = Somewhat untrue for me  3 = Neutral  4 = True for me  5 = Very true for me  Not applicable |
| - I want to change my practice to reduce GHG contributions. | - Ik wil mijn werkwijze veranderen om de uitstoot van broeikasgassen te verminderen. | - Ik wil mijn handelen veranderen om de bijdrage aan broeikasgas te verminderen. | - Ik wil mijn werkwijze veranderen om de uitstoot van broeikasgassen te verminderen. | - Ik wil mijn werkwijze veranderen om de uitstoot van broeikasgassen te verminderen. | - I want to change my practises to reduce greenhouse gases emissions | - I want to change my ways of working to reduce the emission of greenhouse gases. |
| - I want to teach patients/clients/community members about how climate change impacts health. | - Ik wil patiënten/cliënten/leden van de gemeenschap leren hoe klimaatverandering de gezondheid beïnvloedt. | - Ik wil patiënten/cliënten/burgers leren hoe klimaatverandering gezondheid beïnvloedt. | - Ik wil patiënten/cliënten/mensen in de samenleving leren hoe klimaatverandering de gezondheid beïnvloedt. | - Ik wil patiënten/cliënten/mensen in de samenleving leren hoe klimaatverandering de gezondheid beïnvloedt. | - I want to teach patients/ clients/ people in the community how climate change affects health | - I want to teach patients/clients/people in the community how climate change impacts health. |
| - I want to prepare for health impacts of climate change at my workplace. | - Ik wil me voorbereiden op de gezondheidseffecten van klimaatverandering op mijn werkplek. | - Ik wil mij voorbereiden op de impact van klimaatverandering op gezondheid op mijn werkplek. | - Ik wil mij op mijn werkplek voorbereiden op de gevolgen van klimaatverandering op de gezondheid. | - Ik wil mij voorbereiden op de gezondheidsgevolgen van klimaatverandering op mijn werkplek. | - I want to prepare for the health impacts of climate change in my workplace. | - At my workplace, I want to prepare myself for the impact of climate change on health. |
| **Motivation 2:** The following are reasons I am ***motivated*** to address climate change: (Check all that apply) | **Motivatie 2:** De volgende redenen **motiveren** mij om klimaatverandering aan te pakken: (Vink alles aan wat van toepassing is) | **Motivatie 2:** De volgende aspecten zijn redenen waarom ik **gemotiveerd**ben om me bezig te houden met klimaatverandering: (Kruis alles aan wat van toepassing is). | **Motivatie 2:** De volgende redenen **motiveren** mij om klimaatverandering aan te pakken: (Vink alles aan wat van toepassing is). | **Motivatie 2:** De volgende redenen **motiveren** mij om klimaatverandering aan te pakken: (Vink alles aan wat van toepassing is). | **Motivation 2:** The following reasons **motivate** me to address climate change: (Tick all that apply) | **Motivation 2:** The following reasons **motivate** me to address climate change: (Tick all that apply). |
| - Personal experience with nature | - Persoonlijke ervaring met de natuur | - Persoonlijke beleving van de natuur | - Persoonlijke natuurbeleving | - Persoonlijke natuurbeleving | - Personal nature experience. | - Personal nature experiences |
| - Religious/faith/spiritual | - Religieus/geloof/spiritueel | - Religieus/geloof/spiritueel | - Religieus/geloof/spiritueel | - Religieus/geloof/spiritueel | - Religious/ faith/ spiritual. | - Religious/belief/spiritual |
| - Health impacts | - Gezondheidseffecten | - Gezondheidseffecten | - Gevolgen voor de gezondheid. | - Gevolgen voor de gezondheid. | - Health implications. | - Health impact. |
| - Financial costs | - Financiële kosten | - Financiële kosten | - Financiële kosten. | - Financiële kosten. | - Financial costs. | - Financial cost. |
| - Social justice, inequity | - Sociale rechtvaardigheid, ongelijkheid | - Sociale rechtvaardigheid, ongelijkheid | - Sociale rechtvaardigheid, ongelijkheid. | - Sociale rechtvaardigheid, ongelijkheid. | - Social justice, inequality. | - Social justice, inequality. |
| - The future | - De toekomst | - De toekomst | - De toekomst. | - De toekomst. | - The future. | - The future. |
| - Clean air and water | - Schone lucht en water | - Schone lucht en schoon water | - Schone lucht en schoon water. | - Schone lucht en schoon water. | - Clean air and clean water. | - Clean air and clean water. |
| - Loss of property | - Verlies van eigendom | - Verlies van eigendom |  | - Verlies van eigendom |  |  |
| - Increasing severity of weather | - Toenemende ernst van het weer | - Toenemende weersextremen | - Toenemende weersextremen. | - Toenemende weersextremen. | - Increasing weather extremes. | - Increasing weather extremes. |
| - Worse wildfires | - Ergere bosbranden | - Heftigere natuurbranden | - Ergere natuurbranden. | - Ergere natuurbranden. | - Worse wildfires. | - More severe wildfires. |
| - Infectious disease | - Infectieziekten | - Infectieziekten | - Infectieziekten. | - Infectieziekten. | - Infectious diseases. | - Infectious diseases. |
| - Sea level rise | - Stijging van de zeespiegel | - Zeespiegelstijging | - Zeespiegelstijging. | - Zeespiegelstijging. | - Rising of sea level | - Sea level rise. |
| - My family | - Mijn familie | - Mijn familie | - Mijn familie. | - Mijn familie. | - My family. | - My family. |
| - To help create healthy communities. | - Om gezonde gemeenschappen te creëren | - Helpen bij het realiseren van gezonde gemeenschappen | - Om te helpen een gezonde samenleving te realiseren. | - Om een gezonde gemeenschap helpen te creëren. | - To help make a healthy society a reality. | - To help to realize a healthy community. |
| - To live within my ecological footprint | - Om binnen mijn ecologische voetafdruk te leven | - Leven binnen mijn ecologische voetafdruk | - Om binnen mijn ecologische voetafdruk te leven. | - Om binnen mijn ecologische voetafdruk te leven. | - To live within my ecological footprint. | - To live within my ecological footprint. |
| - To protect the planet | - Om de planeet te beschermen | - Beschermen van de planeet | - Om de planeet te beschermen. | - Om de aarde te beschermen. | - To protect the planet. | - To protect the planet. |
| - Professional obligation | - Professionele verplichting | - Professionele verplichting | - Professionele verplichting | - Professionele verantwoordelijkheid | - Professional obligation. | - Professional duty. |
| - To reduce climate impacts of my work and workplace | - Om de klimaatimpact van mijn werk en werkplek te verminderen | - Verminderen van de impact van mijn werk en werkplek op het klimaat | - Om de klimaatimpact van mijn werk en werkplek te verminderen | - Om de klimaatimpact van mijn werk en werkplek te verminderen | - To reduce the climate impact of my work and workplace. | - To reduce the impact on the climate that my job and workplace have. |
| - Not applicable - I’m not motivated to take action. | - Niet van toepassing – ik ben niet gemotiveerd om actie te ondernemen | - Niet van toepassing – Ik ben niet gemotiveerd om actie te ondernemen | - Niet van toepassing – ik ben niet gemotiveerd om actie te ondernemen | - Niet van toepassing – ik ben niet gemotiveerd om actie te ondernemen | - Not applicable – I am not motivated to take action. | - Not applicable – I am not motivated to take any action. |
| - Other:______ | - Anders:_____ | - Anders:.. | - Anders… | - Anders… | - Otherwise:… | - Other … |
| **Motivation 3:** The following are reasons I do **NOT** address climate change to the extent I would like: (Check all that apply) | **Motivatie 3:** De volgende redenen zorgen ervoor dat ik klimaatverandering niet aanpak in de mate waarin ik zou willen: (Vink alles aan wat van toepassing is) | **Motivatie 3:** De volgende aspecten zijn redenen waarom ik me **NIET**met klimaatverandering bezighoud in de mate waarin ik dat graag zou willen: (Kruis alles aan wat van toepassing is). | **Motivatie 3:** De volgende redenen zorgen ervoor dat ik klimaatverandering **NIET** aanpak in de mate waarin ik zou willen: (Vink alles aan wat van toepassing is). | **Motivatie 3:** De volgende redenen zorgen ervoor dat ik klimaatverandering **NIET** aanpak in de mate waarin ik zou willen: (Selecteer alles aan wat van toepassing is). | **Motivation 3:** The following reasons cause me **NOT** to address climate change to the extent I would like (Tick all that apply). | **Motivation 3:** For the following reasons, I do **NOT** address climate change as much as I would like:  (Tick all that apply). |
| - Loss of jobs | - Verlies van banen | - Baanverlies | - Baanverlies. | - Afname werkgelegenheid. | - Job loss. | - Job loss. |
| - National security | - Nationale veiligheid | - Nationale veiligheid | - Nationale veiligheid. | - Nationale veiligheid. | - National security. | - National security. |
| - It costs too much. | - Het kost te veel | - Het kost te veel. | - Het kost te veel. | - Het kost te veel. | - It costs too much. | - It costs too much. |
| - I am overwhelmed. | - Ik ben overweldigd | - Ik ben overweldigd. | - Ik ben overweldigd. | - Ik ben overweldigd. | - I am over whelmed | - I feel overwhelmed. |
| - It is too complex. | - Het is te complex | - Het is te complex. | - Het is te complex. | - Het is te complex. | - It is too complex. | - It is too complex. |
| - Humans can’t reduce climate change. | - Mensen kunnen klimaatverandering niet verminderen | - Mensen kunnen de klimaatverandering niet verminderen. | - Mensen kunnen klimaatverandering niet verminderen. | - Mensen kunnen klimaatverandering niet verminderen. | - Humans cannot mitigate climate change. | - Humans are not able to reduce climate change. |
| - I don’t know enough about climate change. | - Ik weet niet genoeg over klimaatverandering | - Ik weet niet genoeg over klimaatverandering. | - Ik weet niet genoeg over klimaatverandering. | - Ik weet niet genoeg over klimaatverandering. | - I don't know enough about climate change | - I have insufficient knowledge about climate change. |
| - I don’t know what to do. | - Ik weet niet wat ik moet doen | - Ik weet niet wat te doen | - Ik weet niet wat ik moet doen. | - Ik weet niet wat ik kan doen. | - I don't know what to do. | - I would not know what to do. |
| - Political views | - Politieke opvattingen | - Politieke standpunten. | - Politieke opvattingen. | - Politieke opvattingen. | - Political views. | - Political views. |
| - I have more pressing concerns. | - Ik heb urgentere zorgen | - Ik heb dringendere zorgen. | - Ik heb dringendere zorgen. | - Ik heb dringendere zorgen. | - I have more pressing concerns. | - I have more urgent concerns. |
| - It is not convenient. | - Het is niet handig | - Het is niet gemakkelijk. | - Het komt niet goed uit. | - Het komt niet goed uit. | - It is not convenient. | - This is not a good time. |
| - I choose to spend time on other important issues. | - Ik kies ervoor om tijd te besteden aan andere belangrijke kwesties | - Ik kies ervoor mijn tijd aan andere belangrijke zaken te besteden. | - Ik kies ervoor om tijd te besteden aan andere belangrijke zaken. | - Ik kies ervoor om tijd te besteden aan andere belangrijke zaken. | - I choose to spend time on other important things. | - I choose to spend time on other relevant matters. |
| - I am too busy. | - Ik heb het te druk | - Ik heb het te druk. | - Ik heb het te druk. | - Ik heb het te druk. | - I'm too busy. | - I am too busy. |
| - I’m not confident to act. | - Ik heb niet genoeg zelfvertrouwen om te handelen | - Ik ben niet zelfverzekerd genoeg om te handelen. | - Ik ben niet zelfverzekerd genoeg om te handelen. | - Ik voel me niet zelfverzekerd genoeg om iets te doen. | - I am not confident enough to act. | - I am not confident enough to do something. |
| - Not Applicable-I do address climate change to the extent I would like. | - Niet van toepassing – ik pak klimaatverandering aan in de mate waarin ik zou willen | - Niet van toepassing – Ik houd me bezig met klimaatverandering in de mate waarin ik dat zou willen. | - Niet van toepassing – Ik pak klimaatverandering aan in de mate waarin ik zou willen. | - Niet van toepassing – Ik pak klimaatverandering aan in de mate waarin ik zou willen. | - Not applicable – I am addressing climate change to the extent I would like. | - Not applicable – I address climate change as much as I would like to. |
| - Not Applicable-I do not want or intend to address climate change. | - Niet van toepassing – ik wil of ben niet van plan om klimaatverandering aan te pakken | - Niet van toepassing – Ik wil me niet, of ben niet van plan om me bezig te houden met klimaatverandering | - Niet van toepassing – Ik wil niet, of ben niet van plan om klimaatverandering aan te pakken. | - Niet van toepassing – Ik wil niet, of ben niet van plan om klimaatverandering aan te pakken. | - Not applicable – I do not want, or do not plan to address climate change | - Not applicable – I do not want to, or do not intend to address climate change. |
| - Other:_____________ | - Anders:___________ | - Anders:... | - Anders…. | - Anders…. | - Otherwise: … | - Other … |

**Behavior**

| English [Original] | Dutch [Translation 1] | Dutch [Translation 2] | Dutch [Synthesis] | Dutch [Expert Committee consolidation] | English [Back translation 1] | English [Back translation 2] |
| --- | --- | --- | --- | --- | --- | --- |
| **Behavior 1:** How often do you perform the following behaviors at ***home:***    **Behavior Scale** (for 1 and 2)  1 = Never  2 = Rarely  3 = Sometimes  4 = Often  5 = Always | **Gedrag 1:** Hoe vaak vertoon je de volgende gedragingen thuis?    **Gedrag schaal** (voor 1 en 2)  1 = Nooit  2 = Zelden  3 = Soms  4 = Vaak  5 = Altijd | **Gedrag 1:** Hoe vaak vertoont u het volgende gedrag **thuis**:    **Gedrag Schaal** (voor 1 en 2):  1 = Nooit  2 = Zelden  3 = Soms  4 = Vaak  5 = Altijd | **Gedrag 1:** Hoe vaak vertoont u het volgende gedrag ***thuis***?    **Gedrag schaal** (voor 1 en 2):  1 = Nooit  2 = Zelden  3 = Soms  4 = Vaak  5 = Altijd | **Gedrag 1:** Hoe vaak vertoont u het volgende gedrag ***thuis***?    **Gedrag schaal** (voor 1 en 2):  1 = Nooit  2 = Zelden  3 = Soms  4 = Vaak  5 = Altijd | **Behaviour1:** How often do you exhibit the following behaviours ***at home?***    **Behaviour scale** (for 1 and 2):  1 = Never  2 = Rarely  3 = Sometimes  4 = Often  5 = Always | **Behaviour 1:** How often do you engage in the following behaviours ***at home***?  **Behaviour scale** (for 1 and 2):  1 = Never  2 = Seldom  3 = Sometimes  4 = Often  5 = Always |
| - Use non-fossil fuel-based energy sources (such as purchase wind or solar energy, geo-thermal, buy energy offsets, etc.) | - Gebruik energiebronnen die niet op fossiele brandstoffen zijn gebaseerd (zoals het kopen van wind- of zonne-energie, geothermische energie, het kopen van energiecompensaties enz.) | - Gebruiken van niet-fossiele brandstof-gebaseerde energiebronnen (zoals wind of zonne-energie,  aardwarmte, het kopen van energie compensaties, etc) | - Energiebronnen gebruiken die niet op fossiele brandstoffen zijn gebaseerd (zoals het inkopen van wind- of zonne-energie, aardwarmte, het kopen van energiecompensaties, etc.) | - Energiebronnen gebruiken die niet op fossiele brandstoffen zijn gebaseerd (zoals het inkopen van wind- of zonne-energie, aardwarmte, CO2 compenseren het kopen van energiecompensaties, etc.). | - Using energy sources not based on fossil fuels ( such as purchasing wind or solar energy, geothermal energy, buying energy offsets etc.). | - Use energy sources that are not derived from fossil fuels (like purchasing wind or solar energy, geothermal heat, buying energy compensations, et cetera) |
| - Conserve energy (such as use energy efficient appliances, keep moderate temperature settings, turn off lights and electronics, etc.) | - Energie besparen (zoals het gebruik van energiezuinige apparaten, gematigde temperatuurinstellingen aanhouden, lichten en elektronica uitschakelen, enz.) | - Besparen van energie (zoals gebruiken van energiezuinige apparaten, lager instellen van de thermostaat, uitdoen van lichten en electronica, etc.) | - Energie besparen (zoals het gebruik van energiezuinige apparaten, de thermostaat lager instellen, lichten en elektronica uitschakelen, etc.) | - Energie besparen (zoals het gebruik van energiezuinige apparaten, de thermostaat lager instellen, lichten en elektronica uitschakelen, etc.) | - Saving energy ( such as using energy-efficient  appliances, lowering the thermostat, turning off lights and electronic appliances). | - Save energy (e.g., using energy-efficient supplies, turning down the thermostat, turning off lights and appliances, et cetera) |
| - Use less gasoline (drive fuel-efficient vehicles, reduce unnecessary trips, bike-walk, etc.) | - Gebruik minder benzine (rijd brandstofefficiënte voertuigen, verminder onnodige ritten, fietsen, lopen, enz.) | - Minder benzine gebruiken (rijden in energiezuinige voertuigen, verminderen van onnodige reizen, fietsen-wandelen, etcera) | - Minder benzine gebruiken (rijden in energiezuinige voertuigen, verminderen van onnodige ritten, fietsen, lopen etc.) | - Minder brandstof gebruiken (rijden in energiezuinige voertuigen, verminderen van onnodige ritten, fietsen, lopen etc.). | - Using less petrol ( driving energy-efficient vehicles, reducing unnecessary trips, cycling, walking etc.). | - Use less fuel (driving fuel-efficient cars, reducing the number of unnecessary trips, cycling, walking, et cetera) |
| - Reduce waste (buy less, reuse more, recycle and compost more) | - Afval verminderen (minder kopen, meer hergebruiken, recyclen en meer composteren) | - Verminderen van afval (minder kopen, meer hergebruiken, recyclen en composteren) | - Afval verminderen (minder kopen, meer hergebruiken, recyclen en meer composteren) | - Afval verminderen (minder kopen, meer hergebruiken, recyclen en meer composteren) | - Reduce waste ( buy less, reuse more, recycle and compost more). | - Reduce waste (buying less, re-using more, recycling and increasing composting) |
| - Choose foods that require fewer resources to grow/produce (local, seasonal, fewer animal products, less packaging) | - Kiezen van voedingsmiddelen die minder middelen nodig hebben om te groeien/produceren (lokaal, seizoensgebonden, minder dierlijke producten, minder verpakking) | - Kiezen van voedingsmiddelen die minder grondstoffen nodig hebben om te groeien of geproduceerd te worden (lokaal, van het seizoen, minder dierlijke producten, minder verpakkingsmateriaal) | - Voedingsmiddelen kiezen die minder grondstoffen nodig hebben om te groeien/produceren (lokaal, seizoensgebonden, minder dierlijke producten, minder verpakkingsmateriaal | - Voedingsmiddelen kiezen die minder grondstoffen nodig hebben om te groeien/produceren (lokaal, seizoensgebonden, minder dierlijke producten, minder verpakkingsmateriaal | - Choose foods that require fewer resources to grow/ produce (local, seasonal, less animal products, less packaging). | - Choose foods that need less resources to grow/produce (locally grown, seasonal, less animal-derived foods, less packaging material) |
| **Behavior 2:** How often do you do the following behaviors at **work**: (If you do not work or volunteer in a professional setting, please skip this question) | **Gedrag 2:** Hoe vaak vertoon je de volgende gedragingen op het **werk**: (als je niet werkt of vrijwilligerswerk doet in een professionele omgeving, sla deze vraag over) | **Gedrag 2:** Hoe vaak vertoont u het volgende gedrag op het **werk**: (Als u niet werkt of  vrijwilliger bent in een professionele setting, sla dan deze vraag over). | **Gedrag 2:** Hoe vaak vertoont u het volgende gedrag op het **werk**: (als u niet werkt óf geen vrijwilligerswerk doet in een professionele setting, sla deze vraag over). | **Gedrag 2:** Hoe vaak vertoont u het volgende gedrag op het **werk**: (als u niet werkt óf geen vrijwilligerswerk doet in een professionele setting, sla deze vraag over). | **Behaviour 2:** How often do you exhibit the following behaviours at **work:** ( if you do not work or volunteer in a professional setting, skip this question). | **Behaviour 2:** How often do you show the following behaviour at **work**:  (skip this question if you do not work or volunteer in a professional environment) |
| - Conserve energy (such as turn off lights and electronics, etc.) | - Energie besparen (zoals het uitdoen van lampen en elektronica, enz.) | - Besparen van energie (zoals uitdoen van lichten en elektronica, etc.) | - Energie besparen (zoals lichten en elektronica uitschakelen, etc.) | - Energie besparen (zoals lichten en elektronica uitschakelen, etc.) | - Saving energy ( such as turning off lights and electronic devices) | - Save energy (e.g., turn off lights and electronic devices, et cetera) |
| - Commute to work using active (bike, walk), shared, or public transportation. | - Woon-werkverkeer met actieve (fietsen, lopen), gedeelde of openbare vervoersmiddelen. | - Woon-werk verkeer via actief (fietsen, wandelen), gedeeld (carpoolen), of openbaar vervoer. | - Woon-werkverkeer via actief vervoer (fietsen, lopen), gedeeld (carpoolen) of openbaar vervoer. | - Woon-werkverkeer via actief vervoer (fietsen, lopen), gedeeld (carpoolen) of openbaar vervoer. | - Commuting through active transportation (cycling, walking), sharing (carpooling) or public transportation | - Commuting by active transport (cycling, walking), shared transport (car-sharing), or public transport. |
| - Reduce waste (plastic, paper, linen, clinical supplies, etc.) | - Afval verminderen (plastic, papier, linnen, klinische benodigdheden, enz.) | - Verminderen van afval (plastic, papier, linnen, klinische benodigdheden, etc.) | - Afval verminderen (plastic, papier, linnen, klinische benodigdheden, etc.) | - Afval verminderen (plastic, papier, linnen, klinische benodigdheden, etc.) | - Reduce waste (plastics, paper, linen, clinical supplies etc.). | - Reduce waste (plastic, paper, linen, clinical supplies, et cetera). |
| - Ask leaders at your workplace to support policies, products and/or processes that create fewer greenhouse gases (GHGs) | - Vraag leiders op je werkplek om beleid, producten en/of processen te ondersteunen die minder broeikasgassen (BKG's) uitstoten. | - Het vragen aan leiders op uw werkplek om beleid, producten en/of processen te ondersteunen die minder broeikasgassen uitstoten. | - Leiders op uw werkplek vragen om beleid, producten en/of processen te ondersteunen die minder broeikasgassen uitstoten. | - Leidinggevenden op uw werkplek vragen om beleid, producten en/of processen te ondersteunen die minder broeikasgassen uitstoten. | - Asking leaders in you workplace to support policies, products and/ or processes that emit fewer greenhouse gases. | - Ask workplace leaders to support policy, products and/or processes that emit less greenhouse gases. |
| **Behavior 3:** How often do you communicate (in-person, phone, email, letter, etc.) about climate change and health with these groups or individuals?      **Behavior scale** (for 3)  1 = Never  2 = Yearly  3 = 2-3 times per year  4 = Monthly  5 = Weekly | **Gedrag 3:** Hoe vaak communiceer je (in persoon, telefonisch, per email, per brief, enz.) over klimaatverandering en gezondheid met de volgende groepen of individuen?    **Gedrag schaal** (voor 3)  1 = Nooit  2 = Jaarlijks  3 = 2-3 keer per jaar  4 = Maandelijks  5 = Wekelijks | **Gedrag 3:** Hoe vaak communiceert u (persoonlijk, telefonisch, via e-mail, brief, etc.) met deze groepen of individuen over klimaatverandering en gezondheid?    **Gedrag schaal**(voor 3)  1 = Nooit  2 = Jaarlijks  3 = 2-3 keer per jaar  4 = Maandelijks  5 = Wekelijks | **Gedrag 3:** Hoe vaak communiceert u (in levende lijve, telefonisch, per e-mail, per brief, etc.) over klimaatverandering en gezondheid met de volgende groepen of individuen?    **Gedrag schaal** (voor 3):  1 = Nooit  2 = Jaarlijks  3 = 2-3 keer per jaar  4 = Maandelijks  5 = Wekelijks | **Gedrag 3:** Hoe vaak communiceert u (persoonlijk, telefonisch, per e-mail, per brief, etc.) over klimaatverandering en gezondheid met de volgende groepen of individuen?    **Gedrag schaal** (voor 3):  1 = Nooit  2 = Jaarlijks  3 = 2-3 keer per jaar  4 = Maandelijks  5 = Wekelijks | **Behaviour 3**: How often do you communicate (in person, by phone, email, letter etc.) about climate change and health with the following groups or individuals?    **Behaviour scale** (for 3):  1 = Never  2 = Annual  3 = 2-3 times a year  4 = Monthly  5 = Weekly | **Behaviour 3:** How often do you communicate (personally, by phone, by e-mail, by letter, et cetera) about climate change and health with the following groups or individuals?    **Behaviour Scale** (for 3):  1 = Never  2 = Yearly  3 = 2-3 times a month  4 = Monthly  5 = Weekly |
| - Professionally (e.g. colleagues, patients, clients) | - Professioneel (e.g. collega's, patiënten, cliënten) | - Professioneel (bijv. collega's, patiënten, cliënten) | - Professioneel (bijv. collega's, patiënten, cliënten). | - Professioneel (bijv. collega's, patiënten, cliënten). | - Professional (e.g. colleagues, patients, clients). | - Professional (e.g., colleagues, patients, clients). |
| - Personally (friends, family, neighbors) | - Persoonlijk (vrienden, familie, buren) | - Persoonlijk (vrienden, familie, buren) | - Persoonlijk (vrienden, familie, buren). | - Privé (vrienden, familie, buren). | - Personal (friends, family, neighbours) | - Personal (friends, relatives, neighbours). |
| - Elected officials or community leaders | - Verkozen functionarissen of gemeenschapsleiders | - Gekozen ambtenaren of gemeenschapsleiders. | - Verkozen ambtenaren of leiders in de samenleving | - Volksvertegenwoordigers of maatschappelijke partijen. | - Elected officials or leaders in society | - Elected officials or leaders in the community. |

| Final: Is there anything else you would like to add? | Laatste vraag: Is er nog iets wat je wilt toevoegen? | Tot slot: Is er iets anders dat u zou willen toevoegen? | Tot slot: Is er nog iets dat u wilt toevoegen? | Tot slot: Is er nog iets anders dat u wilt toevoegen? | Finally, is there anything you would like to add? | Finally, is there something you would like to add? |
| --- | --- | --- | --- | --- | --- | --- |

**Supplementary file IV.** *List of questions presented to the developers of the CHANT.*

| Original | ** = post-interview questions* |
| --- | --- |
| Awareness 1: Please indicate your level of familiarity with the following evidence-based statements. |  |
| - Climate change increases the likelihood of adverse health conditions such as heat stroke, asthma exacerbation, Lyme disease, and others. | **We are having trouble interpreting the use of ‘adverse health conditions’ in this question. What is your interpretation of this? The reason for this is that it became clear during our interview sessions that some participants made distinctions between actual diagnoses and experienced symptoms.* |
| Awareness 2: I have heard about climate change from these sources (check all that apply). |  |
| - Professional Organizations | *What is the meaning of this source exactly? Are these professional organizations that focus on sustainability specifically, are these nursing-specific professional organizations or just any professional organizations?* |
| Experience 2: The Centers for Disease Control identify several health conditions that are worsened by climate change. For each group, how often are you seeing these conditions? | **We noticed that some participants interpreted this question in relation to climate change and some participants didn’t. How should this question be interpreted? To give an example: Are the participants supposed to describe how often they see the conditions in relation to climate change or just overall?* |
| Motivation 1: Health care contributes approximately 8.5% of Greenhouse Gases (GHGs) in the US. Please indicate how true the following statements are *for you.* | *What is the meaning of this question exactly? During our discussions we had some difficulty with the meaning of this question. Because statistical information is presented, we figured that the question could also mean: 'to what extent is the evidence accepted by you (do you believe the evidence?) and therefore causing you to become motivated to do the following?'* |
| - I want to change my practice to reduce GHG contributions. | *What meaning does 'practice' have in this sentence? We thought it could mean both: 'the way of working' or 'workplace'.* |
| - I want to prepare for health impacts of climate change at my workplace. | *What is emphasized in this question exactly? We are unsure what exactly is emphasized in this question; 'workplace' or 'preparing'.*   - *Does it mean: 'I want to* ***prepare*** *for health impacts of climate change at my workplace'. Or does it imply that the participant wants to prepare his/her* ***workplace*** *to be ready for the health impacts of climate change?* |
| Motivation 2: The following are reasons I am *motivated* to address climate change: (Check all that apply) |  |
| - To help create healthy communities. | *What is the meaning of this exactly? We are trying to find a good equivalent for this due to cultural differences. What does a community mean in your context?* |
| Motivation 3: The following are reasons I do NOT address climate change to the extent I would like: (Check all that apply) |  |
| - Loss of jobs | **We noticed that a lot of participants did not fully understand what was meant by ‘loss of jobs’. How should this be interpreted? We thought it would mean something in the lines of: Working on climate change might make it so that other people may lose their jobs due to change in processes/technologies. We also thought it could mean that ‘you’ as a person might lose your job.* |
| - Political views | **Does this mean the participant's political views or the current ruling political views (current president)? A few participants would describe the current ruling political party as a reason that climate change is not addressed (less funding for example).* |
| - I’m not confident to act. | *What does this mean exactly? Does it ask about the participant's level of self-confidence or whether the participant is convinced enough to confidently take action?* |
| Behavior 3: How often do you communicate (in-person, phone, email, letter, etc.) about climate change and health with these groups or individuals? |  |
| - Elected officials or community leaders | *What is the meaning of this exactly? We are trying to find a good equivalent for this due to cultural differences. Currently we translated this as 'representatives or social parties'. Would this be correct?* |

**Supplementary file V**. *Modification indices and exploratory factor analysis.*


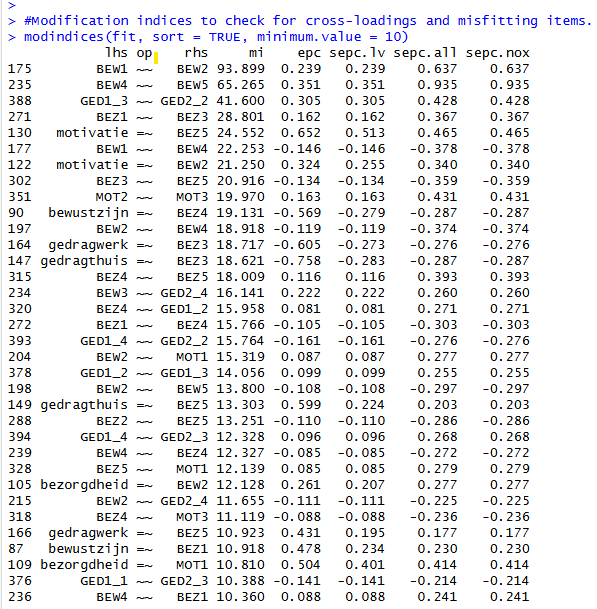


*EFA - Eigen values*


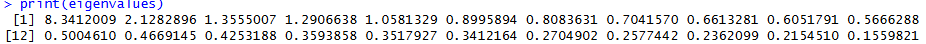


*EFA – Scree plot*

*
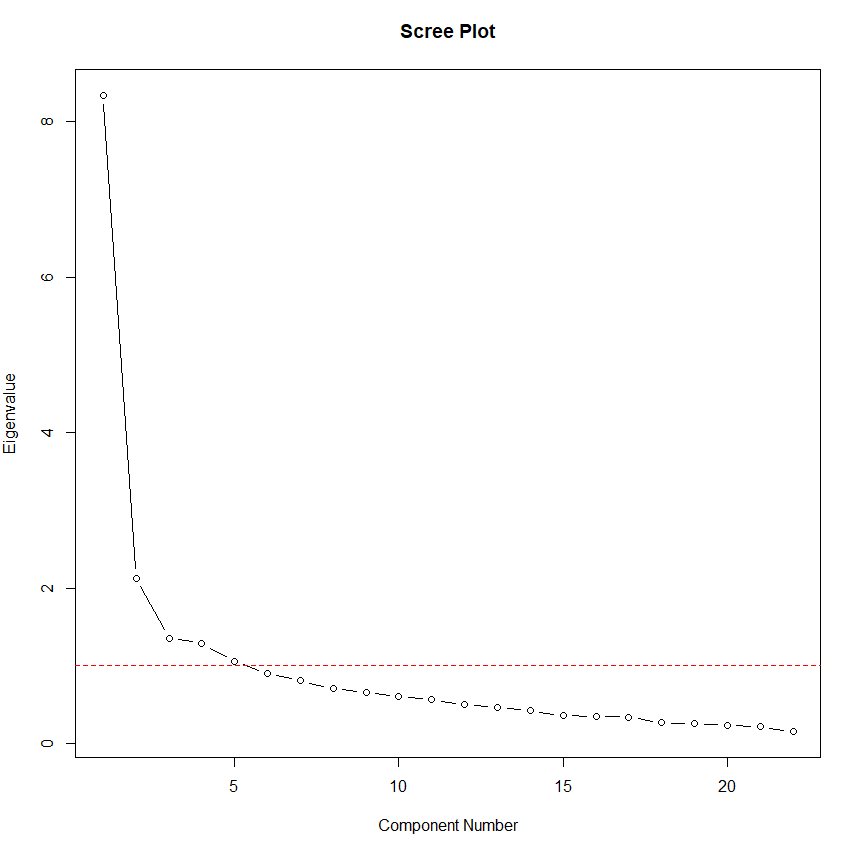
*

*EFA with Varimax rotation*


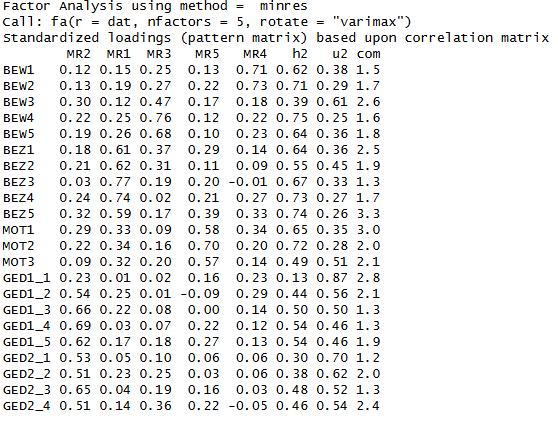


*EFA with Oblique rotation*


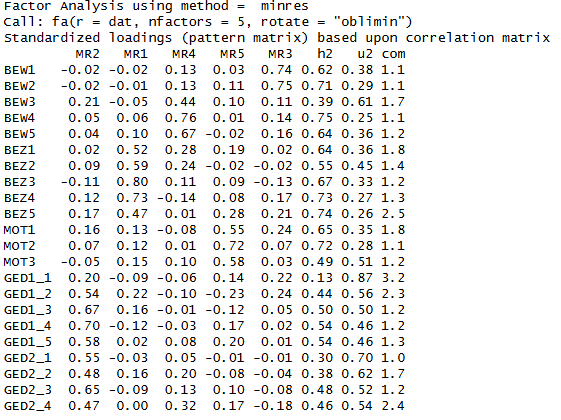

Supplement: Supplementary file 1 [file mmc1.docx]
